# Tunable room-temperature ferromagnet using an iron-oxide and graphene oxide nanocomposite

**DOI:** 10.1038/srep11430

**Published:** 2015-06-23

**Authors:** Aigu L. Lin, J. N. B. Rodrigues, Chenliang Su, M. Milletari, Kian Ping Loh, Tom Wu, Wei Chen, A. H. Castro Neto, Shaffique Adam, Andrew T. S. Wee

**Affiliations:** 1 NUS Graduate School of Integrative Sciences and Engineering, National University of Singapore,28 Medical Drive, Singapore 117456; 2 Centre for Advanced 2D Materials and Graphene Research Centre, Faculty of Science, National University of Singapore, 6 Science Drive 2, Singapore 117546; 3 Department of Physics, Faculty of Science, National University of Singapore, 2 Science Drive 3, Singapore 117542; 4 Department of Chemistry, Faculty of Science, National University of Singapore, 3 Science Drive 3, Singapore 117543; 5 Materials Science and Engineering, King Abdullah University of Science and Technology (KAUST), Thuwal, 23955-6900, Saudi Arabia; 6 Yale-NUS College, 16 College Ave West, Singapore 138527

## Abstract

Magnetic materials have found wide application ranging from electronics and memories to medicine. Essential to these advances is the control of the magnetic order. To date, most room-temperature applications have a fixed magnetic moment whose orientation is manipulated for functionality. Here we demonstrate an iron-oxide and graphene oxide nanocomposite based device that acts as a tunable ferromagnet at room temperature. Not only can we tune its transition temperature in a wide range of temperatures around room temperature, but the magnetization can also be tuned from zero to 0.011 A m^2^/kg through an initialization process with two readily accessible knobs (magnetic field and electric current), after which the system retains its magnetic properties semi-permanently until the next initialization process. We construct a theoretical model to illustrate that this tunability originates from an indirect exchange interaction mediated by spin-imbalanced electrons inside the nanocomposite.

Manipulating the properties of a ferromagnet by means other than a magnetic field has had tremendous impact on technology. The most prominent example of this is the spin-transfer torque mechanism predicted by Slonczewski^1^ and by Berger^2^, in which a spin-polarized electrical current transfers angular momentum to the ferromagnet and switches its orientation. Magnetic memories based on this mechanism (ST-MRAM) are already commercially available, are non-volatile, have better energy efficiency, and are more readily scalable to smaller devices than most conventional memory^3^. Another example is magnetoelectric (or multiferroic) materials^4^,^5^,^6^ where the magnitude of the magnetization can be controlled by an electric field. For room temperature operation, magnetoelectric materials are made by engineering heterostructures combining ferroelectric and ferromagnetic materials that are coupled by strain at their interface. Such materials could also have application in low latency memory. Yet another promising mechanism to control ferromagnetic properties includes controlling the transition temperature of thin ferromagnetic films using an electric field^7^,^8^,^9^ —this exploits the sensitivity of magnetic properties to the electronic carrier density tuned by the field effect.

In this work, we report on a nanocomposite material that allows for its magnetic properties to be controlled in a new way. The material (discussed in detail below) is a nanocomposite of graphene oxide and iron-oxide nanoparticles. We show that using an initialization procedure involving a magnetic field and a spin-polarized electric current, we can controllably set the magnetic moment and transition temperature of the ferromagnet that then remains stable even after the current and magnetic fields are switched off. Operating at room temperature, this gives an example of a system where the magnetism itself can be switched on or off depending on the current and magnetic fields that are applied during the initialization step. The mechanism relies on an electron spin-imbalance generated during initialization, that gives rise to an electron mediated ferromagnetic coupling between the iron nanoparticles. Starting from a simplified microscopic Hamiltonian, we show theoretically that the coupling is indeed ferromagnetic, and provide Monte Carlo simulations for the dependence of the transition temperature on spin-imbalance that is consistent with experimental observations. This ability to electrically turn on and off the magnetization might enable applications in nonvolatile memories with novel operation modes and using easy processable materials, as well as hybrid devices integrating tunable electric and magnetic components.

The device consists of a nanocomposite of partially reduced (between 18% and 20%) and highly defective graphene oxide^10^ mixed up with iron-oxide (FeO/Fe_3_O_4_ complex) core shell structure nanoparticles to which one attaches two pinned ferromagnetic cobalt electrodes whose configuration is driven by an external magnetic field (see Fig. 1). The nanoparticles are in a canted ferrimagnetic alpha-phase and carry magnetic moments of approximately 3 to 5 *μ*_*B*_ (and typical diameter of 6.5–9.5 nm)^11^. At room-temperature, due to their small dimension, the nanoparticles are in a superparamagnetic state having their magnetic moment thermally flipping between their two easy axis directions. The graphene oxide contains a high concentration of nanovoids, vacancies and adatoms which carry magnetic moments that are the origin of the paramagnetic response observed in the graphene oxide sheets^10^ without the iron-oxide nanoparticles. The graphene oxide is partially reduced and thus the carbon atoms whose p^*z*^-orbitals are not passivated can be regarded as sites where electrons can localize. The hopping electrons moving through the nanocomposite can hop between these sites through variable range hopping—see supplementary information.

The mixture is strongly disordered: there are nanoparticles of different sizes and thus different magnetic moments, whose position and easy axis orientation is random; the partially reduced graphene oxide flakes are also randomly positioned and oriented; thus, from the point of view of a hopping electron, the sites it can occupy are randomly positioned having random onsite energies. Using the external magnetic field to drive the magnetic orientation of the two cobalt electrodes, a spin-imbalance can be generated in the nanocomposite’s population of hopping electrons whenever an electric current flows across the device at room temperature (throughout the text, we refer to this as the *initialization process*). The source electrode spin-polarizes the current entering the nanocomposite, while the drain electrode acts as a filter allowing electrons with one spin orientation to preferentially leak out the nanocomposite. When the electrodes are in an anti-parallel (parallel) configuration they generate (destroy) a spin-imbalance in the population of hopping electrons of the system. An antiferromagnetic PtMn layer pins the cobalt electrodes magnetic orientation via exchange bias so that their magnetization will only be flipped by a sufficiently strong magnetic field.

If no electric current is passed across the device, the nanocomposite is paramagnetic for all tested temperatures. This indicates that the nanocomposite’s magnetic moments (both from the iron oxide nanoparticles and from the defective graphene oxide) are essentially independent. The nanocomposite remains paramagnetic when a spin-unpolarized electric current is passed across it. However, using ferromagnetic electrodes to inject a spin-polarized current into the nanocomposite, the system can be made to undergo a ferromagnetic transition depending on the particular magnetic configuration of the electrodes. Of practical interest is the fact that this configuration can be controlled by an external magnetic field. Has shown in Fig. 2, the initialization is done with two accessible knobs: a potential bias driving an electric current that is injected into the nanocomposite through two ferromagnetic electrodes; and an external magnetic field (with a magnitude of the order of tens of mT) driving the magnetic configuration of the electrodes. These two knobs determine the device’s magnetic properties which remain stable for as long as we have measured it (several weeks) after the electric current and magnetic field are turned off.

We argue below that the spin-polarized current injected into the nanocomposite generates a spin-imbalance on the population of hopping electrons of the nanocomposite. These spin-polarized hopping electrons effectively couple the magnetic moments of the iron-oxide nanoparticles (and of the graphene oxide) through an indirect exchange interaction reminescent of the RKKY interaction^12^,^13^,^14^. The strength of this interaction depends on the degree of spin-imbalance in the population of hopping electrons: a greater spin-imbalance gives rise to a stronger interaction. The strongly disordered nanocomposite implies that the disorder average of this interaction is exponentially damped and effectively ferromagnetic. Thus, it will effectively behave as a disordered array of Heisenberg moments constrained to point around their randomly oriented easy axis and give rise to magnetic clusters that, depending on the *initialization* process, may lead to long range magnetic order. In what follows we will show step-by-step the evidence and reasoning leading to this picture.

We first discuss the spin-dependent electronic transport properties of the device. An electrical current was injected on the device through the ferromagnetic electrodes while an external magnetic field was applied to the system to drive the configuration of the electrodes. We have measured the electrical resistance of the device while gradually varying the strength of the magnetic field. Figure 3(a) shows the result of such a measurement.

Starting from the electrodes in a parallel configuration (*B*_ext_ = −0.6 T) we first increase the magnetic field (forward sweep, black curve). At 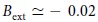 T there is an increase in resistance caused by the switching of one electrode resulting in an antiparallel configuration [see lower panel of Fig. 3(b)]. This is the well known giant magnetoresistance effect (GMR)^15^,^16^ except that our high resistance values suggest that we are in the variable range hopping regime (VRH) rather than the metallic one^17^,^18^. More interesting is a second (and larger) jump in the resistance that occurs when there is no change in the electrode’s configuration (between arrow’s 2 and 3 in Fig. 3). This second jump is related to the ferromagnetic transition that is the main result of this work. Further increasing *B*_ext_, we then observe the expected drop in resistance (between arrow 3 and 4) when the second electrode switches orientation. This corresponds to both the usual GMR effect and the loss of the spin-imbalance required for the ferromagnetic state, which brings the nanocomposite back to a paramagnetic state. The exact same sequence is observed for the backward sweep (red curve, labeled 5–8) where the region 7 corresponds to the range of *B*_ext_ for which we find ferromagnetism in the nanocomposite.

In order to investigate the origin of this effect, we have probed the device’s magnetic properties after having passed an electric current through it at different external magnetic fields to *initialize* it. We have checked that whenever the magnetic field is such that the sample resistance is small [i.e., it is either *B*_ext_ < 0.02 T or *B*_ext_ > 0.05 T—see Fig. 3(a)], the nanocomposite is paramagnetic. However, whenever the *initialization* process is performed with an external magnetic field in the range of high sample resistance (i.e. *B*_ext_ ∈ [0.02, 0.05] T), the nanocomposite is found to be in a ferromagnetic state. This confirms that the sharp jumps in the electrical resistance of the device are related to the ferromagnetic transition occurring in the nanocomposite. Note that the orientation of the electrodes is necessary for this transition, since it only occurs when they are anti-aligned. As mentioned before, a strong spin-imbalance in the population of hopping electrons is only generated for the anti-parallel electrodes’ configuration.

Capacitance is a direct measure of the spin-imbalance generated in the nanocomposite. When the device is in the anti-parallel configuration the measured capacitance increases with increasing magnetic field [see Fig. 2(b)]. The peak capacitance increases from 2 nF at *B*_ext_ ≈ 0.02 T to 9 nF when the applied field is *B*_ext_ ≈ 0.04 T. Further increase of *B*_ext_ did not lead to a noticeable change to the peak capacitance value. In contrast, whenever the device is in its parallel configuration, the measured capacitance is invariably one or two orders of magnitude smaller than that measured for the anti-parallel electrodes’ configuration.

The comparison between the capacitance and the magnetization measurements indicates that the nanocomposite becomes ferromagnetic at room-temperature whenever the capacitance increases above a critical value of 6 nF. The sharp decrease of capacitance once the drain electrode is reversed at *B*_ext_ ≈ 0.05 T confirms the intuitive picture that the trapped spin-polarized charges are released when the electrodes become parallel. Moreover, the fact that the system transitions back to the paramagnetic state, confirms that it is the spin-imbalance that controls the magnetic state of the nanocomposite.

Finally, the temperature dependence of magnetization was measured for several samples *initialized* under different magnetic fields - see Fig. 2(a). The transition temperature (*T*_*b*_) was observed to be strongly affected by the nanocomposite’s spin-imbalance, as indicated by the sample capacitance: when the capacitance is 9 nF, *T*_*b*_ ≈ 317 K; *T*_*b*_ decreases to 309 K and 276 K when the capacitance decreases to 6 nF and 2 nF respectively; whenever the capacitance is 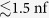, no ferromagnetic ordering is observed even when the temperature is decreased to 10 K. Figure 2 clearly demonstrates that the two external knobs present during the *initialization* process (*B*_ext_ and *V*_ext_) control the spin-imbalance in the population of hopping electrons of the nanocomposite and the magnetic properties of the system.

To understand this ferromagnetic transition we first estimate the direct magnetostatic interaction between the iron-oxide nanoparticles. We find that this is several orders of magnitude smaller than *k*_*B*_*T*_room_ implying that it can be ruled out as the origin of the magnetism in this system. This explains why the system always remains paramagnetic when no current is passed through it. Moreover, the localized electron states are necessary to explain the origin of the ferromagnetism, since no ferromagnetism is observed in experiments without the partially reduced graphene oxide, e.g. when it is replaced with highly conducting graphene or strongly reduced graphene oxide.

The next logical step is to include a Zeeman-like coupling between the hopping electrons and the iron-oxide nanoparticles. This will give rise to an effective interaction between the nanoparticles mediated by the sea of spin-polarized hopping electrons [without spin-imbalance, this is reminescent of the well known Ruderman-Kittel-Kasuya-Yosida (RKKY) interaction^12^,^13^,^14^]. Basically, an electron in the vicinity of one nanoparticle will retain information on its orientation that will then be seen by the other nanoparticles. Estimates of the magnitude of this coupling prove difficult due to uncertainties in several parameters of the system. However, reasonable estimates for material parameters (see supplementary information) suggest that the energy scale of these mediated interactions can be of the order of *k*_*B*_*T*_room_. This is therefore the most plausible explanation for the observed phenomena. In what follows we take *J*_0_ to be the scale of the coupling between the hopping electron and the nanoparticle. This will be an input into the theoretical calculations.

With such a mechanism in mind we can write an effective microscopic Hamiltonian governing a system of hopping electrons with spin and localized iron-oxide magnetic moments. We use Ising moments for the model and do not believe that the behavior would be qualitatively different for a different choice—see supplementary information. The Hamiltonian reads


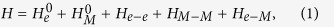


where the 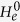 (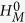) stands for the part governing free electrons (Ising moments), *H*_*e*−*e*_ (*H*_*M*−*M*_) stands for the part containing electron-electron interactions (dipole-dipole interactions), while *H*_*e*−*M*_ stands for the part containing the Zeeman interaction between hopping electrons and Ising moments. In what follows we put aside the terms 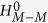 and 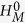, not relevant to the following computations, and, for the sake of simplicity, disregard the term *H*_*e*−*e*_. Following the spirit of RKKY^12^,^13^,^14^ interaction, considering terms in *H* that flip the spin of the hopping electrons, and employing several simplifications to the calculation (see supplementary information), one finds that integrating out the hopping electrons’ degrees of freedom gives rise to the following effective Hamiltonian for the Ising moments (see supplementary information)


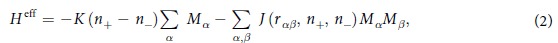


where *M*_*α*_ stands for the magnetic moment indexed by *α* (expressed in terms of Bohr magnetons, *M*_*α*_ = *μ*_*B*_*m*_*α*_*λ*_*α*_, with *λ*_*α*_ = ±1), *r*_*αβ*_ stands for the distance between the two magnetic moments indexed by *α* and *β*, the constant *K* reads *K*  ≡  *J*_0_*μ*_0_*μ*_*B*_*A*, while *n*_*σ*_ stands for the average density of hopping electrons with spin *σ* = +,−. The first term therefore acts on each Ising moment as an effective magnetic field generated by the cloud of spin-imbalanced hopping electrons. The second term is a local indirect exchange interaction term between different Ising moments, with the RKKY-like exchange parameter *J*(*r*, *n*_+_, *n*_−_) given by


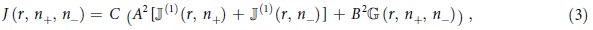


where 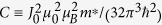 and 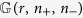 is given by
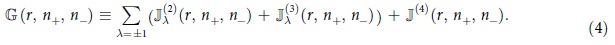
In the above equation the functions 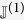, 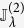, 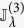 and 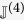 read


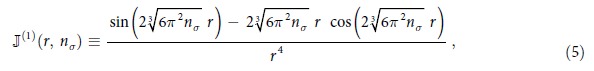



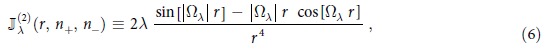



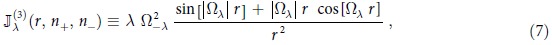



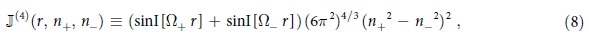


where we have defined 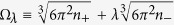. In these expressions *μ*_0_ (*μ*_*B*_) stands for the vacuum permitivity (Bohr magneton), m^*^ for the effective mass of the free hopping electron gas, while *A* (*B*) stands for the amplitude for an electron with spin state *σ* to have its spin unchanged (flipped) when interacting with a nanoparticle.

From Fig. 2(b) we estimate the sample’s average electronic densities, *n*_±_, finding that they are typically small such that first-neighbor interactions are generally ferromagnetic — see supplementary information. Assuming that 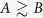 then we conclude that *J*(*r*, *n*_+_, *n*_−_) is minimal for spin-imbalance zero, growing with increasing spin-imbalance—see Fig. 4(a). This is in contrast with the typical RKKY result where no spin-flips of the electrons are considered. Our analytical result explains how the ferromagnetic coupling increases with spin-imbalance explaining the experimental observation that the magnetization vanishes without the spin-imbalance and increases with larger spin-imbalance.

Strong disorder exponentially suppresses the typical value of the RKKY interaction^19^,^20^,^21^ as *J*(*r*, *n*_+_, *n*_−_) → *J*(*r*, *n*_+_, *n*_−_)*e*^−*r*/*ξ*^, where in the metallic case *ξ* is the electron’s mean free path. Since our system is strongly disordered *ξ* should be small, and the exponential suppression essentially kills all longer ranged interactions, such that the only relevant interactions in our system are those comparable with the first-neighbor ones. Therefore all the relevant interactions are ferromagnetic. To compare with the experiment we take *ξ* to be a fitting parameter comparable to the spacing between the nanoparticles.

The experimental results strongly suggest that the first order term in equation (2) is irrelevant when compared with the second order one (see supplementary information). This is perfectly compatible with the theoretical model despite the fact that the effective Hamiltonian—see equation (2)—arises from a series expansion on the electron-nanoparticle interaction. The relative magnitude of the effective Hamiltonian’s first and second order terms is determined by the *external* parameters (*n*_+_ −*n*_−_, *ξ*, *J*_0_, m^*^, *A* and *B*) rather than by the expansion parameter. The parameters used to obtain the results of Fig. 4, yield a second order term at least one order of magnitude greater than the first order one, for the range of spin-imbalances estimated from the experimental results. Accordingly, only the second order term was considered when performing the Monte Carlo simulations.

For typical values of *ξ*, the exponentially damped coupling gives rise to the ordering of the system in magnetic clusters that interact weakly between themselves. Upon decreasing temperature, the magnetic moments inside each cluster start aligning, with different clusters doing so at slightly distinct temperatures. Moreover, as clusters interact weakly, individual clusters will generally have different magnetization directions. As a consequence, the system should not in general present long-range order when temperature is decreased below the *blocking* temperature *T*_*b*_ and this is confirmed in our Monte Carlo simulations — see supplementary information. Similarly, if we remain at a fixed temperature while turning on the exchange interaction (by generating a spin-imbalance in the system), one should not observe long-range order in the system. However, if we start from an ordered state generated, for example, by applying an external magnetic field when the spin-imbalance is being generated (as is done in the experiment), then long-range order should be observed since the nearly independent clusters were from the beginning aligned by the external magnetic field. This is observed in our system: if no magnetic field is applied to the device while the current is flowing across it, no magnetization is observed (see supplementary information for a detailed discussion). In Fig. 4 we show that, when starting from an ordered state, Metropolis Monte Carlo simulations show a transition between an ordered and a disordered state upon variation of temperature. Its blocking temperature depends on the magnitude of the indirect exchange, that we have shown is dependent on the spin-imbalance of hopping electrons.

In conclusion, we have demonstrated a tunable magnet where the iron-oxide and graphene oxide nanocomposite undergoes a paramagnetic to ferromagnetic transition whenever a critical concentration of spin-polarized electrons are trapped within the nanocomposite such that they can generate a sufficiently strong indirect exchange coupling between neighboring iron nanoparticles. This ferromagnetic state is controllable by tuning the spin-imbalance of hopping electrons through the external magnetic field and the potential bias that drives the current across the device during its *initialization* process. Moreover, this state is reversible by elimination of the spin-imbalance, in which case the nanocomposite transitions back to a paramagnetic state. Such artificial composite materials with easily processable components and highly tunable magnetic/transport properties open doors towards constructing high-performance data storage and spintronic devices operating at room temperature.

## Methods

Graphene oxide was synthesized based on the Hummers method. Graphite flakes (3.0 g) were stirred in ice bath. Sodium nitrate (3.0 g) and concentrated sulfuric acid (135 ml) were added into the round-bottom flask. Next, potassium permanganate (18 g) was added slowly over 2 hours. Once the mixture is homogeneous, the solution was transferred to 35 C oil bath and stirred for another 1 hour. A thick paste was formed and deionized (DI) water (240 ml) was added. The mixture was stirred for 1 hour as the temperature was increased to 90 C. Deionized water (600 ml) was added, followed by slow addition of 30% hydrogen peroxide (18 ml) solution. The color of the suspension changed from brown to yellow. The suspension was filtered and washed with 3% HCl solution. It was then repeatedly centrifuged and decanted until the pH of the supernatant is 7. The as-produced graphene oxide was dispersed in 750 ml DI water at a concentration of 0.6 mg/mL^−1^. 3 g of NaOH plate was added into graphene oxide solution (0.1 mol/L). The mixture was refluxed in a round bottom flask under constant magnetic stirring for 1 hour. Subsequently, the based treated GO were separated by centrifuging at 13000 rpm. It was then repeatedly centrifuged and decanted until the pH of the supernatant is 7. The iron oxide nanoparticles were then added to the solution and dispersed with the application of ultrasound for 30 seconds before the solution was spin coated on a silicon dioxide substrate at a speed of 8000 rpm for 30 seconds. We repeated this spin coating process 3 times before thermally reducing the nanocomposite by applying a temperature of 340 K for 15 minutes. Cobalt electrodes 10 nm thick and 200 nm apart were then deposited on the nanocomposite. 20 nm of PtMn was then deposited on one of the cobalt electrodes and was slowly cooled down while an external magnetic field of 0.1 T was applied. This is repeated for the other cobalt electrode but with the external magnetic field applied in the opposite direction. I-V measurements were done after connecting two probes onto the two electrodes using a KEITHLEY Semiconductor Characterization System with voltage varying from 0 to 5 V. The magnetic field was generated using a DEXTER Adjustable Pole Electromagnet (Model # 1607037) and was varied from 0 T to 0.6 T for each I-V measurement. VRH data obtained from the Quantum Design Physical Property Measurement System (PPMS) measurements where the electrical resistance is measured at a fixed magnetic field strength and the temperature is gradually decreased at intervals of 5 K from 298 K to 210 K under a constant applied voltage of 0.5 V. The PPMS is also used to measure the change in electrical resistance at a fixed temperature but under varying magnetic field strengths from 0 to 0.06 T. The magnetic characterization of the device is done by a superconducting quantum interference device (SQUID) magnetometer.

In order to investigate the magnetic ordering of the 3D disordered Ising model arising from the integration of the electronic degrees of freedom, we have performed Monte Carlo simulations using our own implementation of Metropolis single spin-flip algorithm^22^ and Wolf’s cluster algorithm^23^.

## Additional Information

**How to cite this article**: Lin, A. L. *et al.* Tunable room-temperature ferromagnet using an iron-oxide and graphene oxide nanocomposite. *Sci. Rep.* **5**, 11430; doi: 10.1038/srep11430 (2015).

## Supplementary Material

Supplementary Information

## Figures and Tables

**Figure 1:**
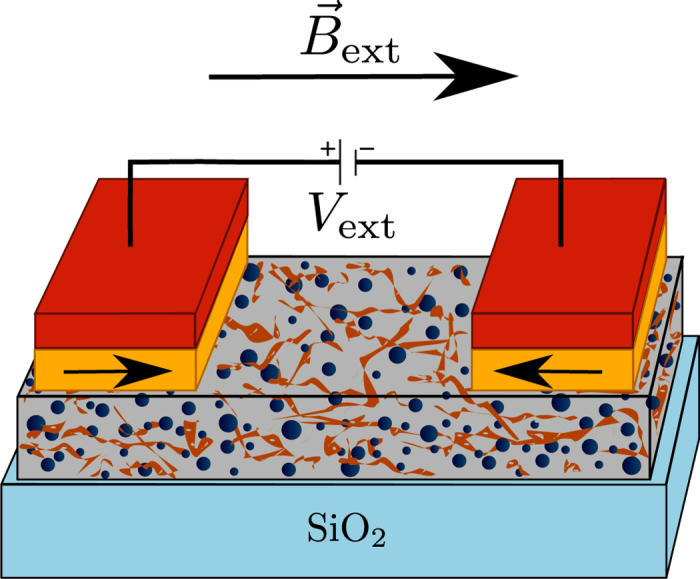
Schematic of the device geometry and nanocomposite composition. The gray box represents the nanocomposite, with the blue spheres representing the iron-oxide nanoparticles and the brown strips representing the highly defective graphene oxide layers. The nanocomposite’s thin film is deposited on top of a silicon dioxide substrate (in light blue). Two cobalt ferromagnetic electrodes (yellow) are placed on top of the nanocomposite. For zero applied magnetic field, these are pinned in an anti-parallel configuration by PtMn layers (in red).

**Figure 2:**
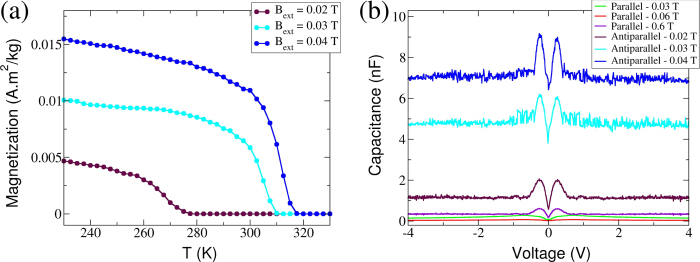
Magnetization and capacitance for different initialization processes. (**a**) Magnetization as a function of temperature for samples *initialized* with different *B*_ext_. The transition temperature can be made to vary from 276 K (*B*_ext_ = 0.02 T) to 317 K (*B*_ext_ = 0.04 T). (**b**) Capacitance measurement for several different *B*_ext_ corresponding to distinct electrodes configuration (see text and Fig. 1 for details).

**Figure 3:**
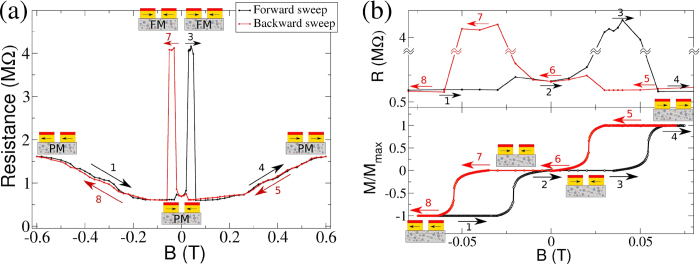
Device’s electrical properties. (**a**) Device’s electrical resistance in terms of the *B*_ext_. **(b)** Ferromagnetic pinned electrodes response to an external magnetic field (bottom) and a blow up of the resistance data in the same field range (top). Measurements reveal two distinct jumps in resistance, one corresponding to the giant magnetoresistance and the other due to a ferromagnetic transition.

**Figure 4:**
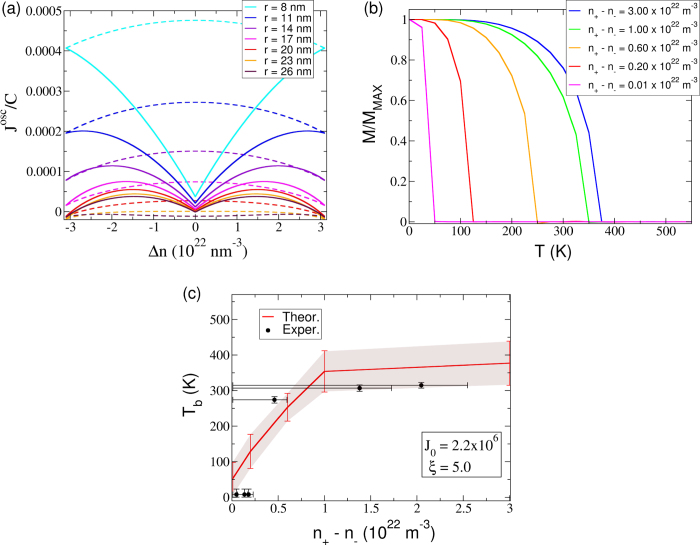
Exchange parameter and Monte Carlo Simulations of a disordered 3D Ising model with exponential decaying RKKY interactions. (**a**) Plot of the oscillating part of the exchange parameter [see equation (3)] for several distances between nanoparticles. The constant *C* dividing *J*^osc^ is the leading factor in equation (3). We plot both the case where no electron’s spin-flips are allowed (dashed curves) and the case where these are allowed (full curves). (**b**) Magnetization in terms of the temperature for different strengths of the indirect exchange coupling. (**c**) Comparison between the experimental and theoretical blocking temperature in terms of the strength of the spin-imbalance (the theoretical fitting parameters used were *J*_0_ = 2.2 × 10^6^ and *ξ* = 5 nm). The Monte Carlo results of panels (b) and (c) were obtained for simulations (with 79507 Ising moments) starting from a highly ordered state (see supplementary information) that use Metropolis algorithm. They explore the phase space region in the vicinity of the global energy minimum, and indicate that the system can show long range order if the clusters are initially aligned by an external magnetic field.
