# Supplementary material for: Tunable room-temperature ferromagnet using an iron-oxide and graphene oxide nanocomposite
Source: Sci Rep. 2015 Jun 23;5:11430. doi: 10.1038/srep11430 (PMC4477240; doi:10.1038/srep11430)
Supplement: Supplementary Information [file srep11430-s1.pdf]

# Supplementary Information

## Tunable room-temperature ferromagnet using an iron-oxide and graphene oxide nanocomposite

Aigu L. Lin, J. N. B. Rodrigues, Chenliang Su, M. Milletari, Kian Ping Loh,
Tom Wu, Wei Chen, A. H. Castro Neto, Shaffique Adam and Andrew T. S. Wee

## I. ADDITIONAL EXPERIMENTAL INFORMATION

### The nanocomposite

The nanocomposite's graphene oxide was thermally reduced by approximately 18% to 20%. Its electrical resistance was observed to decrease from an initial magnitude of $(1.15 \pm 0.03)$ MΩ to $(0.94 \pm 0.04)$ MΩ after the reduction process.

The left panel of Supplementary Fig. S1 shows a TEM micrograph of the nanocomposite where it is possible to see its strongly disordered nature with the nanoparticles randomly positioned and the graphene oxide flakes placed in between them. From this figure we can estimate that the typical distance between nanoparticles is roughly $\approx 12$ nm.

### The transport properties of the device

In the right panel of Supplementary Fig. S1 we plot the device's resistance (in logarithmic scale) in terms of the fourth root of the temperature to show the variable range hopping nature of the electronic transport in the nanocomposite.

### The magnetization measurements

Each one of the curves in main text's Fig. 2(a), showing the temperature dependence of the nanocomposite's magnetization (at zero-field) were obtained by *initializing* the system (at room temperature) with the indicated $B_{\text{ext}}$. After *initialization* the temperature was decreased to $T \approx 230$ K and from there the system's magnetization was recorded while increasing temperature up to $T \approx 330$ K. Whenever the *initialization* was done with $0 \text{ T} \lesssim B_{\text{ext}} \lesssim 0.015$ T, no magnetization was observed upon temperatures of $T \approx 10$ K. Note that the several curves of Fig. 2(a) of the main text were obtained for different samples, since after measuring the sample magnetization up to $T \approx 330$ K the graphene oxide layers present in the nanocomposite were strongly reduced and thus the nanocomposite properties irreversibly changed (see discussion of Section III).

The left panel of Supplementary Fig. S2 shows the ferromagnetic response of the nanocomposite after *initialization* with a magnetic field of $B_{\text{ext}} = 0.03$ T (orange curve) and $B_{\text{ext}} = 0.04$ T (green curve). Its right panel shows several room-temperature magnetization values obtained for an ensemble of successive *initialization* processes done (at room temperature) with different magnetic fields $B_{\text{ext}}$. This set of measurements was done using the same sample.

### Capacitance measurements

The capacitance measurements presented in main text's Fig. 2(b) were performed during the *initialization* process, i. e. at the same time that the magnetic field was being applied to the device (to drive its electrode's configuration) and the electric current was flowing across it. In sub-Section I A we discuss a simple picture for this capacitance measurement that allows us to estimate the spin-imbalance of the hopping electrons' population. A detailed discussion of the ingredients involved in the generation and retention of the spin-imbalance is done in Section III.

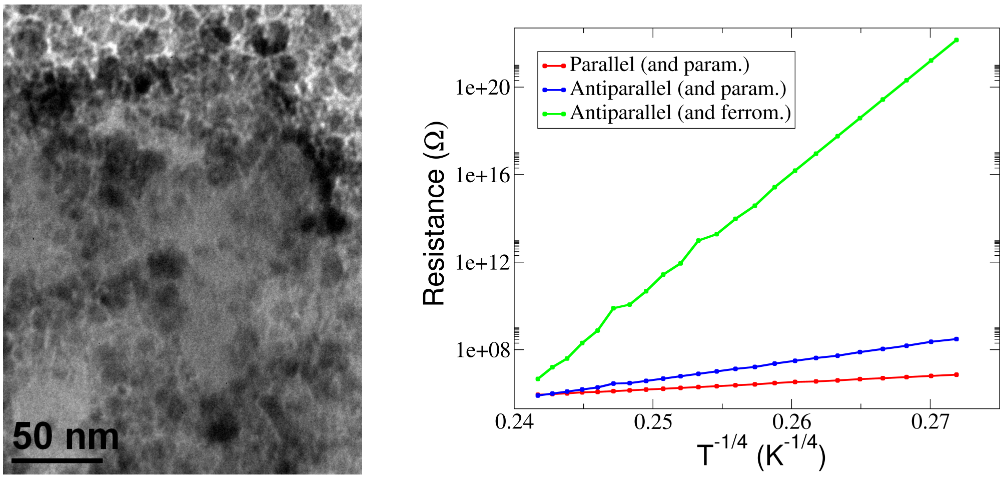


**Supplementary Figure** S1. **Left:** TEM micrograph of the nanocomposite, where we can see the nanoparticles (spheroidal structures) surrounded by the graphene oxide flakes (bright structures). From it we can roughly estimate that the typical nanoparticles' radius is $\approx 5$ nm, while the distance between their centers is roughly $\approx 12$ nm. **Right:** Plots showing the variable range hopping character of the electronic transport in the nanocomposite, $R(T) = R_0 \exp\left[(T_M/T)^{1/4}\right]$. The Mott temperature $T_M$ in each of the cases reads: $T_M \approx 2.41 \times 10^4$ K (red); $T_M \approx 1.48 \times 10^9$ K (blue); $T_M \approx 1.92 \times 10^{12}$ K (green).

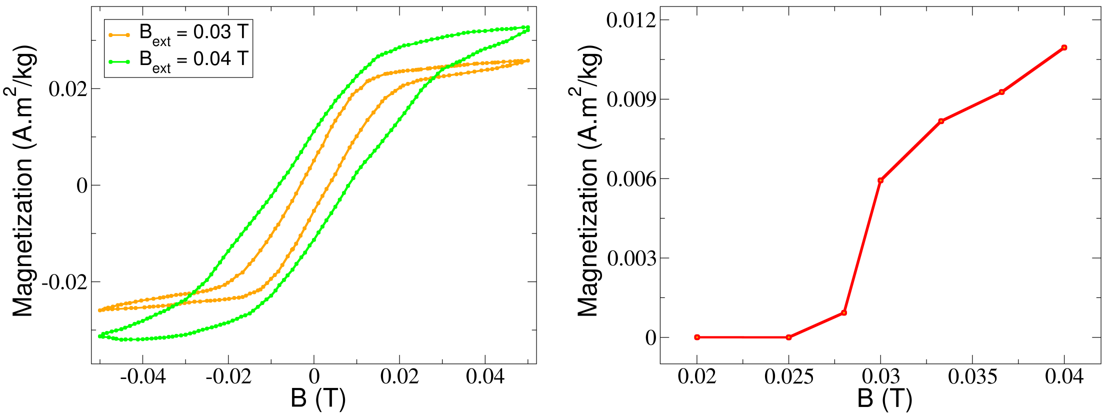


**Supplementary Figure** S2. **Left:** Hysteretic response of the nanocomposite after *initialization* with a spin-polarized current and an external magnetic field of $B_{\rm ext} = 0.03$ T (orange curve) and of $B_{\rm ext} = 0.04$ T (green curve). **Right:** Room-temperature magnetization in terms of the magnetic field applied during the *initialization* process for a given sample that was successively *initialized* (at room temperature) with different magnetic fields.

## Estimates

We estimate several parameters and energy scales of the system, such as: the typical density of the nanocomposite, the average magnitude of the iron-oxide magnetic moments and the spin-imbalances associated with each measured capacitance (see I A); the energy scale of the magnetostatic interaction between two iron-oxide nanoparticles (see I B); the magnitude of the indirect exchange interaction mediated by the hopping electrons (see I C).

### A. Nanocomposite's density, magnetic moments' magnitude and spin-imbalance

To estimate the typical density of the nanocomposite, we note that it is deposited on top of the $SiO_2$ substrate by a spin-coating process which gives rise to a disc-like structure with typical thickness of 150 nm (believed to be nearly uniform) and some hundreds of micrometers of radius. The typical weight of the samples is of the order of $\mu$g. As an example, one of the samples weights $M \approx 1.01\,\mu$g and has a radius of $\approx 1200\mu$m, i.e., its density reads $\rho \approx 1.5 \times 10^6$ g/m$^3$, a value that is compatible with an estimate of the density taking into account the density of each of the nanocomposite's components: $Fe_2O_3$ nanoparticles, graphene oxide, toulene and spacer.

The magnitude of each $Fe_2O_3$ nanoparticle magnetic moment, $m$, depends both on the nanoparticle's size (typical diameter of 6.5-9.5 nm) and on its microscopic ordering ($\alpha$-phase nanoparticles with a ferrimagnetic canted ordering). The value usually assumed for their typical magnetic moment is in the range 3.2-3.6$\mu_B$. But we can still try to estimate it from the measurements made in the system. From the blue curve of main text's Fig. 2(a) (i.e. *initialization* done with $B_{\rm ext} = 0.04$ T) we can conservatively estimate the saturation magnetization of the nanocomposite to be $M_S \approx 2.2 \times 10^{-2}$ A.m$^2$/kg. Using the nanocomposite's mass density, $\rho \approx 1.5 \times 10^6$ g/m$^3$, and the volume per nanoparticle [from Supplementary Fig. S1(a) we estimate the typical distance between nanoparticles to be $\approx 12$ nm] $V_{\rm box} \approx 1.7 \times 10^{-24}$ m$^3$, we estimate the typical magnetic moment associated with such volume to be $\bar{M}_{\rm box} \approx 6.1\ \mu_B$. As only the nanoparticle and the graphene oxide flakes carry magnetic moments, we can estimate that the typical magnetic moment associated with each nanoparticle is $\approx 5.1\ \mu_B$, while that of the graphene oxide contained in that volume is $\approx 1.0\ \mu_B$.

To estimate the spin-imbalance in the hopping electrons' population we adopt the simplistic picture where the device acts as a *spin capacitor*[1–4]: the source electrode spin-polarizes the current entering the nanocomposite, while the drain electrode acts as a filter allowing electrons with the spin state aligned with its magnetization to leak out of the nanocomposite but not those with an opposite one. Anti-parallel (parallel) electrodes generate (destroy) a spin-imbalance in the population of hopping electrons of the nanocomposite. Within such a picture we can roughly estimate the spin-imbalance from the capacitance measurement by $\Delta n \equiv n_+ - n_- = CV/(e\mathbb{V})$, where $C$ is the measured capacitance, $V$ the applied bias voltage, $e$ the electron charge and $\mathbb{V}$ the sample's volume. For the three curves in main text's Fig. 2(b) corresponding to the anti-parallel configuration of the electrodes, $B_{\rm ext} = 0.04$ T, $B_{\rm ext} = 0.03$ T and $B_{\rm ext} = 0.02$ T, the peak capacitance of the device at $V \approx 0.25$ V is approximately 9 nF, 6 nF and 2 nF respectively. Therefore, the corresponding estimated spin-imbalances respectively read $\Delta n \approx 2.04 \times 10^{22}$, $\Delta n \approx 1.38 \times 10^{22}$ and $\Delta n \approx 0.46 \times 10^{22}$ electrons/m$^3$. This rules out Pauli paramagnetism as the phenomenon behind the ferromagnetic state since the overall maximal magnetic moment contributed by the hopping electrons (when such spin-imbalances are at play) is typically two orders of magnitude smaller than the measured nanocomposite's magnetization. Similarly, when the electrodes are parallel aligned [see main text's Fig. 2(b)], the curves obtained with $B_{\rm ext} = 0.03$ T, $B_{\rm ext} = 0.06$ T and $B_{\rm ext} = 0.6$ T, show a peak capacitance (at $V \approx 0.7$ V, $V \approx 0.75$ V and $V \approx 0.25$ V) of approximately 0.3 nF, 0.07 nF and 0.6 nF respectively. Therefore, the corresponding estimated spin-imbalances read $\Delta n \approx 1.80 \times 10^{21}$, $\Delta n \approx 4.90 \times 10^{20}$ and $\Delta n \approx 1.36 \times 10^{21}$ electrons/m$^3$. Note that these estimates for the spin-imbalance are upper bounds, since we expect that the spin-imbalance generated during the *initialization* process relaxes with time due to thermal activated electron spin-flipping – see Section III.

### B. The magnetostatic interaction

In order to estimate the value of the energy associated with the magnetostatic interaction between two iron-oxide nanoparticles, we neglect all the shape and finite size effects playing a role in the interaction between two nanoparticles. We consider, in first order approximation, that it can be well described by the classical dipole-dipole interaction. The

energy of such interaction is given by $U_{\mathrm{MS}} = -\mu_0\, \mathbf{m}_1 \cdot \big(3\mathbf{r}(\mathbf{m}_2 \cdot \mathbf{r})/r^5 - \mathbf{m}_2/r^3\big)/(4\pi)$, where $\mathbf{m}_1$ and $\mathbf{m}_2$ stand for the magnetic moment vectors of the two nanoparticles, while $\mathbf{r}$ stands for the distance separating them. Note that depending on the relative position and orientation of the two magnetic moments, this term may favor ferromagnetism or antiferromagnetism. This interaction energy will at most have a magnitude of $|U_{\mathrm{MS}}| \le \mu_0\, m_1 m_2/(2\pi r^3)$. If the two magnetic moments have a magnitude of $m_{1,2} \approx 4.0\,\mu_B$, and are at a distance of $r \approx 12$ nm, then the magnetostatic interaction energy reads $|U_{\mathrm{MS}}| \approx 1.6 \times 10^{-28} J$. This energy is several orders of magnitude smaller than the room-temperature thermal energy $k_B T_{\mathrm{room}} \approx 4.1 \times 10^{-21}$ J, and thus this interaction can be discarded as the origin of the room-temperature ferromagnetic state. This fact is in accordance with the experimental observation that the nanocomposite is paramagnetic when no current is passed across the system. As was stated above, the experimental observations strongly suggest that it is the spin-imbalance of the hopping electrons' population that is driving the nanocomposite's ferromagnetic transition.

### C. The indirect exchange interaction

To estimate the energy scale of the indirect exchange coupling between the magnetic moments of two iron-oxide nanoparticles, $U_{\mathrm{ex}}$, is tricky since it is not trivial to picture, in a simple manner, an indirect interaction mediated by the hopping electrons. In addition, there are several quantities that are relevant for such an estimate that we can hardly extract from experimental measurements. Nevertheless, by trying to use reasonable system's parameters, in what follows we show that it is indeed possible that the indirect interaction's energy scale is considerably bigger than that of the magnetostatic interaction.

An electron moving through the nanocomposite will first visit a nanoparticle and retain information on its magnetic state, that will later *exhibit* to another nanoparticle that it will subsequently visit. The effective coupling between the two nanoparticles is going to result from the combined effect of the several hopping electrons scattering off both nanoparticles in a given characteristic time scale. Both temperature and disorder are expected to reduce the indirect coupling since they will likely enhance the decoherence of the hopping electron spin state.

We will assume that both the nanoparticle's magnetic moments and the hopping electrons' spin are Ising moments. Furthermore, we will consider that a hopping electron feels a nanoparticle through a Zeeman-like coupling between the electron's spin and the magnetic field generated by the nanoparticle. The energy associated with such an interaction reads $E_{i\alpha} = -\boldsymbol{\mu}_i \cdot \mathbf{B}_\alpha = -\sigma_i \mu_B B_\alpha(\mathbf{r}_i)$, where $\mathbf{B}_\alpha(\mathbf{r}_i)$ stands for the value (at position $\mathbf{r}_i$) of the magnetic field generated by the nanoparticle indexed by $\alpha$ (located at $\mathbf{r}_\alpha$), while $\sigma_i = \pm 1$ stands for the hopping electron's spin and $\mu_B$ is the Bohr magneton. For the sake of simplicity we consider that the magnetic field generated by the nanoparticle only has support inside the nanoparticle, and that it can be approximated by the magnetic field of a uniformly magnetized sphere $B_\alpha(r \le R) = J_0 \mu_0 2 m_\alpha \mu_B/(3V_{nnp})$, where $\mu_0$ stands for the vacuum permitivity, $m_\alpha$ stands for the absolute value (in Bohr magnetons) of the magnetic moment of the nanoparticle positioned at $\mathbf{r}_\alpha$, while $V_{nnp}$ is the volume of the nanoparticle. The inclusion of $J_0$ accounts for the possibility that the scale of the interaction between the electron and the nanoparticle may be stronger than the bare Zeeman coupling. We can simplistically assume that an electron visiting two nanoparticles with $4\mu_B$ magnetic moments, that are separated by 12 nm, will give rise to an indirect interaction with an average energy scale given by $\langle E \rangle \approx J_0 \times 10^{-28}$ J. But we must take into account all the other electrons scattering off the two nanoparticles in the characteristic time of thermal flipping of the nanoparticles ($\approx 1$ ns). Then, a spin-imbalance of $\Delta n \approx 10^{22}$ will correspond to an average of $10^4$ electrons scattering off the two nanoparticles during their characteristic flipping time. In such a case, the typical energy scale of the indirect exchange interaction would be of the order of $U_{\mathrm{ex}} \approx J_0 \times 10^{-24}$ J, a value that is considerably bigger than the magnetostatic interaction energy scale. Still, and if $J_0$ is of the order of $10^3$, then the $U_{\mathrm{ex}}$ becomes comparable to $k_B T_{room}$.

## II. ADDITIONAL THEORETICAL INFORMATION

In this section we will show that by considering a model where hopping electrons and nanoparticles interact through a Zeeman-like coupling, we can qualitatively reproduce the tunability of the magnetic properties of the nanocomposite by the manipulation of the knobs that control the nanocomposite's hopping electrons spin-imbalance. Starting from a microscopic Hamiltonian (for the ensemble of hopping electrons and nanoparticles' magnetic moments) and upon integration of the electronic degrees of freedom we will end up with an effective Hamiltonian governing the nanoparticles' magnetic moments. This Hamiltonian will contain an exchange interaction term that depends on the spin-imbalance of hopping electrons: a stronger spin-imbalance will give rise to a stronger effective coupling between the nanoparticles' magnetic moments. Finally, using Monte Carlo simulations, we will show that the variation of the nanocomposite's ordering temperature with its spin-imbalance qualitatively mimics the experimental observations of higher nanocomposite's ordering temperature for greater spin-imbalance.

### A. Derivation of the effective Hamiltonian

We start by writing the partition function for the system composed of hopping electrons and nanoparticles $Z = \sum_{\{\alpha\}} \sum_{\{i\}} \exp\big(-\beta H'[\{i\},\{\alpha\}]\big)$, where $\{i\}$ ($\{\alpha\}$) indicates a given configuration of the hopping electrons' (nanoparticles') degrees of freedom, while $\beta = 1/(k_B T)$ and $H'[\{i\},\{\alpha\}] \equiv H[\{i\},\{\alpha\}] - \sum_{\sigma=\pm 1} \mu_\sigma N_\sigma$ stands for the system's Hamiltonian. By integrating the electronic degrees of freedom, $\{i\}$, we will write the system's partition function as

$$Z = \sum_{\{\alpha\}} e^{-\beta H_{\mathrm{eff}}[\{\alpha\}]}, \tag{S1}$$

where $H_{\mathrm{eff}}[\{\alpha\}]$ is the effective Hamiltonian governing the nanoparticles.

As pointed out in the main text, we will work with the Hamiltonian $\hat{H} = \hat{H}_e^0 + \hat{H}_{e-M}$, where $\hat{H}_e^0$ is the hopping electron's kinetic term, while $\hat{H}_{e-M}$ contains the interaction between hopping electrons and constrained Heisenberg moments. The hopping electron's kinetic term can be written as $\hat{H}_e^0 = -\sum_{ij}\sum_\sigma\left(\gamma_{ij}\hat{c}^\dagger_{i\sigma}\hat{c}_{j\sigma} + \text{h.c.}\right)$, where $\hat{c}_{i\sigma}$ stands for the (fermionic) operator annihilating an hopping electron with spin $\sigma$ that is siting at position $\mathbf{r}_i$. The $\gamma_{ij}$ stands for the hopping amplitude for an electron sitting at $\mathbf{r}_j$ to hop to the position $\mathbf{r}_i$. Note that as a first approach we do not allow the hopping electrons to flip their spin when hopping between sites, i. e. we impose $\gamma_{ij}^{\sigma'\sigma} \longrightarrow \gamma_{ij}$.

While moving around the nanocomposite the hopping electrons feel the local magnetic fields generated by the nanoparticles. For simplicity, we model such an effect by a local Zeeman-like interaction as follows $\hat{H}_{e-M} = -\sum_{i\alpha} J_0\mu_0\hat{\mathbf{S}}_i\cdot\hat{\mathbf{M}}_\alpha\delta(\mathbf{r_i}-\mathbf{r}_\alpha)$, where $\mu_0$ stands for the vacuum permeability, $\hat{\mathbf{S}}_i$ stands for the angular momentum operator of the hopping electron siting at position $\mathbf{r}_i$, while $\hat{\mathbf{M}}_\alpha$ stands for the angular momentum operator of the nanoparticle siting at position $\mathbf{r}_\alpha$. The delta function enforces the local character of this interaction. The factor $J_0$ sets the scale of the coupling between the hopping electron and the nanoparticle. When writing such an interaction we are assuming the nanoparticle to be a point-like dipole whose magnetic field vanishes everywhere around itself except at the exact position where it is sitting. This corresponds to a first order approximation where all the finite size effects are ignored, which should preserve the general qualitative behavior of the system.

The typical magnitude of the nanoparticles' magnetic moment (between 3 and 5 $\mu_B$) justifies regarding them as classical moments. For simplicity, we consider the nanoparticles' magnetic moments to be Ising (aligned along $\mathbf{e}_z$). In doing such we believe that, despite the mathematical simplifications, the system's qualitative behavior is going to be preserved so that we can still investigate the influence that manipulating the hopping electrons' spin-imbalance has on the system's magnetic ordering. We can indeed consider a more realistic model where the nanoparticles' moments (that are constrained around their randomly oriented easy axis) are assumed to be Ising moments with a randomly aligned easy axis (fluctuations around the nanoparticles' ground states are neglected). In such a case, a generalization of the following calculation can still be done (see end of Section II A 3 for details).

The magnetic moment of the nanoparticle at $\mathbf{r}_\alpha$ is going to be identified as $\mathbf{M}_\alpha = \mu_B m_\alpha\lambda_\alpha\mathbf{e}_z$, where $m_\alpha$ gives the magnetic moment's magnitude in terms of Bohr magnetons, while $\lambda_\alpha = \pm 1$ defines the orientation of the moment. We can express the (Ising) spin operator of a hopping electron sitting at $\mathbf{r}_i$, as $\hat{S}_i^z = \mu_B\sum_{\sigma'\sigma}\hat{c}^\dagger_{i\sigma_i}[\sigma^z]_{\sigma_i\sigma_i'}\hat{c}_{i\sigma_i'} = \mu_B\sigma_i\hat{c}^\dagger_{i\sigma_i}\hat{c}_{i\sigma_i}$, where $\sigma^z$ stands for the corresponding Pauli matrix, while $\sigma_i = \pm 1$ indicates the spin orientation of the electron sitting at $\mathbf{r}_i$. The term describing the electron-nanoparticle interaction can then be written as $\hat{H}_{e-M} = -J_0\mu_0\mu_B^2\sum_{i\alpha}\sum_{\sigma_i} m_\alpha\sigma_i\lambda_\alpha\delta(\mathbf{r_i}-\mathbf{r}_\alpha)t^{(\alpha)}_{\eta_i\sigma_i}\hat{c}^\dagger_{i\eta_i}\hat{c}_{i\sigma_i}$, where by introducing the factor $t^{(\alpha)}_{\eta_i\sigma_i}$, we allow the hopping electrons to flip their spin when they interact with the nanoparticles. This factor stands for the probability amplitude for the electron sitting at the site $\mathbf{r}_i = \mathbf{r}_\alpha$ to have its spin flipped from $\sigma_i$ into $\eta_i$ when interacting with the nanoparticle at position $\mathbf{r}_\alpha$ (electronic spin can be transferred into the nanocomposite). The superscript $\alpha$ in $t^{(\alpha)}_{\eta_i\sigma_i}$ accounts for the fact that these amplitudes depend on the orientation and magnitude of the magnetic moment of the nanoparticle. The full Hamiltonian then reads

$$\hat{H} = -\sum_{ij}\sum_{\sigma_i,\sigma_j}\left(\gamma_{ij}\delta_{\sigma_i\sigma_j}\hat{c}^\dagger_{i\sigma_i}\hat{c}_{j\sigma_j} + \text{h.c.}\right) - J_0\mu_0\mu_B^2\sum_{i\alpha}\sum_{\sigma_i} m_\alpha\sigma_i\lambda_\alpha\delta(\mathbf{r_i}-\mathbf{r}_\alpha)t^{(\alpha)}_{\eta_i\sigma_i}\hat{c}^\dagger_{i\eta_i}\hat{c}_{i\sigma_i}\,. \tag{S2}$$

We now integrate the hopping electrons' degrees of freedom identified by the operators $\hat{c}_{i\sigma_i}$, allowing for electronic spin imbalances through $\hat{H}' \equiv \hat{H} - \sum_\sigma\mu_\sigma\hat{N}_\sigma$, where $\hat{N}_\sigma \equiv \sum_i\hat{c}^\dagger_{i\sigma}\hat{c}_{i\sigma}$. From Eq. (S1) we can write $Z_e^{(\alpha)} = \exp\left[-\beta H^{(\alpha)}_{\text{eff}}\right]$, where $Z_e^{(\alpha)}$ is the partition function for the hopping electrons in a particular (quenched) landscape of the nanoparticles' magnetic moments. In path integral formalism (using Grassmann variables) we can write $Z_e^{(\alpha)} = \int\mathcal{D}(\bar{\psi},\psi)\exp\left(-S[\bar{\psi},\psi;\alpha]\right)$ where $S[\bar{\psi},\psi;\alpha]$ stands for the action in a given quenched landscape of nanoparticles' magnetic moments. The $\mathcal{D}(\bar{\psi},\psi)$ stands for the measure of the path integral, $\mathcal{D}(\bar{\psi},\psi) = \lim_{N\to\infty}\prod_{n=1}^N d(\bar{\psi}^n,\psi^n)$, while the Grassmann fields must satisfy the boundary conditions given by $\psi(0) = -\psi(\beta)$ and $\bar{\psi}(0) = -\bar{\psi}(\beta)$. By substituting the fields by their time Fourier transform we can express the action in the frequency representation as $S[\bar{\psi},\psi;\alpha] = \bar{\Psi}\mathbb{A}\Psi$, where we have used $\Psi = [\Psi^{(1)},\Psi^{(2)},\ldots]^T$ and $\Psi^{(n)} = \left[\psi^{(n)}_{1+},\psi^{(n)}_{1-},\psi^{(n)}_{2+},\psi^{(n)}_{2+},\ldots\right]$, as well as $\mathbb{A} = \text{diag}[\mathbb{A}^{(1)},\mathbb{A}^{(2)},\ldots]$ with $\mathbb{A}^{(n)}_{i\sigma_i,j\sigma_j} = \left[\delta_{ij}\delta_{\sigma_i\sigma_j}(-iw_n-\mu_{\sigma_j}) + \gamma_{ij}\delta_{\sigma_i\sigma_j} + \delta_{ij}\sum_\alpha V_\alpha\sigma_j\lambda_\alpha t^{(\alpha)}_{\sigma_i\sigma_j}\delta(\mathbf{r}_i-\mathbf{r}_\alpha)\right]$, where $V_\alpha = J_0\mu_0\mu_B^2 m_\alpha$ and $\mu_\sigma$ stands for the chemical potential associated with the spin state $\sigma = \pm 1$, while the Matsubara frequencies $w_n = (2n+1)\pi/\beta$ are determined by the boundary conditions for the Grassmann fields, with $n\in\mathbb{Z}$.

Using the result for multidimensional Grassmann gaussian integrals $\int\mathcal{D}(\bar{\psi},\psi)e^{-\Psi^T\mathbb{A}\Psi} = \det\mathbb{A}$, we conclude that the hopping electrons' (quenched) partition function $Z_e^{(\alpha)}$ can be restated as $Z_e^{(\alpha)} = \prod_{w_n}\det\left[\mathbb{A}^{(n)}\right] = \exp\left[\sum_{w_n}\text{Tr}\left[\log(\mathbb{A}^{(n)})\right]\right]$. We can write $\mathbb{A}^{(n)} = -\left[G^{(n)}\right]^{-1} + V$, where $\left[G^{(n)}\right]^{-1}$ stands for the inverse propagator of the free hopping electrons' (with Matsubara frequency $w_n$) and $V$ for the interaction between them and the nanoparticles. Expanding the logarithm in the exponential of $Z_e^{(\alpha)}$, $\log\left(\mathbb{A}^{(n)}\right) = \log\left(-\left[G^{(n)}\right]^{-1}\right) - \left[G^{(n)}V + G^{(n)}VG^{(n)}V/2 + \ldots\right]$ we can rewrite the effective Hamiltonian as

$$\beta H^{(\alpha)}_{\text{eff}} = \sum_{w_n}\left[\text{Tr}\left[\log\left(-\left[G^{(n)}\right]^{-1}\right)\right] - \text{Tr}\left[G^{(n)}V\right] - \text{Tr}\left[\frac{G^{(n)}VG^{(n)}V}{2}\right] + \ldots\right], \tag{S3}$$

where we should remember that the dependence on the nanoparticles' configuration $\{\alpha\}$ of the hopping electrons' partition function $Z_e^{(\alpha)}$ is contained on the interaction terms between the nanoparticles and the hopping electrons, $V = V(\{\alpha\})$. In

this expansion we are only going to be interested in the first and second order terms, since only these terms renormalize the original Hamiltonian's terms, $\hat{H}^0_M$ and $\hat{H}_{M-M}$.

A further simplification of the problem can be done if we both ignore the variable range hopping nature of the electronic dynamics and at the same time consider that the electrons move on a perfect 3-dimensional cubic lattice. We substitute the variable range hopping dynamics by a first-neighbor tight-binding one (and thus $\gamma_{ij} = \gamma$ if $i$ and $j$ are first neighbors). Note that by ignoring the variable range hopping nature of the electronic dynamics and the strong (position and energy) disorder of the sites at which the electrons can sit, we are fundamentally changing the hopping electrons' nature from strongly localized to strongly delocalized. After such a simplification the results of the computations below must be carefully and judiciously interpreted. To do this is equivalent to consider an average over disorder, where the well defined wave-vectors appearing in the following calculations are only meaningful in the scope of the disorder free (i. e. disorder averaged) problem. In order for the results that follow to be more meaningful physically, in the end we are going to substitute the Fermi wave-vectors, $k_F^\sigma$, by the average value of the density of hopping electrons with spin $\sigma$, i. e. $n_\sigma$. Furthermore, we are going to qualitatively account for the strong disorder effects *a posteriori* by including an exponentially damping factor[5–7] (see sub-Section II A 3) in the indirect exchange parameter computed for the clean system.

### 1. *First order term of the effective Hamiltonian*

The zeroth order term of the effective Hamiltonian in Eq. (S3) is just a constant that does not depend on the nanoparticles' configuration. The first order term of the effective Hamiltonian [see Eq. (S3)] is given by $\left[H^{(\alpha)}_{\text{eff}}\right]^{(1)} = -\frac{1}{\beta}\sum_{w_n}\text{Tr}\left[G^{(n)}V\right]$. Expressing it in momentum space and using the fact that the propagator $G^{(n)}$ is diagonal on both the electron's momentum and spin, we can do the sum over Matsubara and the sum in $\mathbf{k}$ corresponding to the trace. We can then write the first order term as

$$\left[H^{(\alpha)}_{\text{eff}}\right]^{(1)} = -J_0\mu_0\mu_B^2\sum_\alpha\left(t^{(\alpha)}_{++}n_+ - t^{(\alpha)}_{--}n_-\right)m_\alpha\lambda_\alpha\,, \tag{S4}$$

where $n_+$ and $n_-$ stand for the average density of hopping electrons with spin $\sigma = +1$ and $\sigma = -1$. We have also explicitly substituted $V_\alpha$ by $V_\alpha = J_0\mu_0\mu_B^2 m_\alpha$.

This term can be interpreted as the response of the nanoparticles' (Ising) magnetic moment to an effective magnetic field generated by the spin-imbalance of the hopping electron gas. Remember that the superscript $\alpha$ in $t^{(\alpha)}_{\sigma\sigma}$, the probability amplitude for an hopping electron with spin $\sigma$ to have its spin conserved when interacting with the nanoparticle at position $\mathbf{r}_\alpha$, indicates that this interaction depends on the state of the nanoparticle. Therefore, and depending on the choice we make for the amplitudes $t^{(\alpha)}_{\eta\sigma}$, we may have different behaviors arising from Eq. (S4) – see Section II A 3.

### 2. *Second order term of the effective Hamiltonian*

Similarly, the second order term of the effective Hamiltonian [see Eq. (S3)] is $\left[H^{(\alpha)}_{\text{eff}}\right]^{(2)} = -\frac{1}{2\beta}\sum_{w_n}\text{Tr}\left[G^{(n)}VG^{(n)}V\right]$, which as the propagator $G^{(n)}$ is diagonal on both the electron's momentum and spin, can be rewritten, after summing over the Matsubara frequencies, as an exchange interaction between two different nanoparticles' Ising magnetic moments

$$\left[H^{(\alpha)}_{\text{eff}}\right]^{(2)} = -\sum_{\alpha,\beta}J(r_{\alpha\beta},k_F^+,k_F^-,\lambda_\alpha,\lambda_\beta)M_\alpha M_\beta\,. \tag{S5}$$

Here the $M_\alpha = \mu_B m_\alpha\lambda_\alpha$ stands for the magnetic moment indexed by $\alpha$ (in Bohr magnetons) aligned along $\lambda_{\alpha,\beta} = \pm 1$, while the exchange parameter reads

$$J(r_{\alpha\beta},k_F^+,k_F^-,\lambda_\alpha,\lambda_\beta) = \frac{J_0^2\mu_0^2\mu_B^2}{2\Omega^2}\sum_{\sigma,\eta=\pm1}\left[\eta\sigma t^{(\alpha)}_{\sigma\eta}t^{(\beta)}_{\eta\sigma}\sum_{\mathbf{k},\mathbf{p}}\left(-\frac{f_\sigma(\mathbf{k})-f_\eta(\mathbf{p})}{E(\mathbf{k})-E(\mathbf{p})-\Delta\mu_{\sigma\eta}}e^{i(\mathbf{p}-\mathbf{k})\cdot\mathbf{r}_{\alpha\beta}}\right)\right], \tag{S6}$$

where $\Delta\mu_{\sigma\eta} \equiv \mu_\sigma - \mu_\eta$ and $f_\sigma(\mathbf{k})$ stands for the Fermi-Dirac distribution function of hopping electrons with spin $\sigma$, i. e. $f_\sigma(\mathbf{k}) \equiv 1/\left[\exp[\beta(E(\mathbf{k})-\mu_\sigma)]+1\right]$.

The above exchange parameter depends on the orientation of the two nanoparticles through the hopping electron flipping amplitudes $t^{(\alpha)}_{\sigma\eta}$ and $t^{(\beta)}_{\eta\sigma}$ and thus we can see it as matrix exchange parameter $J_{\lambda_\alpha\lambda_\beta}$. All the difficulty of computing the exchange parameter is contained on the evaluation of the sums in $\mathbf{k}$ and in $\mathbf{p}$. The expression for the exchange parameter in Eq. (S6) is a sum of four terms arising from the different combinations of $\sigma = \pm1$ and $\eta = \pm1$. For $\eta = \sigma$, i.e., if there is no spin-flipping of the hopping electrons when they interact with the nanoparticles, we end up with the typical RKKY result[8–10]. However, if $\eta = -\sigma$, then we have new terms coming directly from the spin-flip of the electron when it interacts with the nanoparticles[11].

As just mentioned, for $\sigma = \eta$ the sum in $\mathbf{k}$ and $\mathbf{p}$ can be shown to be equal $I_{\sigma\sigma}(r) = \left(m^*\Omega^2/(2(2\pi)^3\hbar^2)\right)\left(\sin(2k_F^\sigma r) - 2k_F^\sigma r\cos(2k_F^\sigma r)\right)/r^4$, where we have assumed both that we are at zero temperature and that the hopping electrons behave as a free electron gas with effective mass $m^*$ and Fermi wave-vector $k_F^\sigma$ (see sub-Section II A 3).

Similarly, the sum in $\mathbf{k}$ and $\mathbf{p}$ when $\eta = -\sigma$ can be shown to be equal to

$$
\begin{aligned}
I_{\sigma,-\sigma}(r) = \frac{m^*\Omega}{4(2\pi)^3\hbar^2}\Bigg[&\left({k_F^\sigma}^2 - {k_F^{-\sigma}}^2\right)^2\left(\mathrm{sinI}\left[(k_F^\sigma + k_F^{-\sigma})r\right] + \mathrm{sinI}\left[(k_F^\sigma - k_F^{-\sigma})r\right]\right)\\
&+ \sum_{\lambda=\pm1}\lambda\left(k_F^\sigma - \lambda k_F^{-\sigma}\right)^2 r^2 \frac{\sin\left[|k_F^\sigma + \lambda k_F^{-\sigma}|r\right] + |k_F^\sigma + \lambda k_F^{-\sigma}|\, r\cos\left[(k_F^\sigma + \lambda k_F^{-\sigma})r\right]}{r^4}\\
&+ 2\sum_{\lambda=\pm1}\lambda\frac{\sin\left[|k_F^\sigma + \lambda k_F^{-\sigma}|r\right] - |k_F^\sigma + \lambda k_F^{-\sigma}|\, r\cos\left[(k_F^\sigma + \lambda k_F^{-\sigma})r\right]}{r^4}\Bigg], \qquad \text{(S7)}
\end{aligned}
$$

where $\mathrm{sinI}[x]$ stands for the sine integral function of $x$.

For simplicity we consider that the hopping electron's spin-flip amplitudes at the nanoparticle positioned at $\mathbf{r}_\alpha$ do not depend on the orientation of the nanoparticle, i.e. $t_{\sigma\eta}^{(\alpha)} = t_{\sigma\eta}\ \forall\alpha$. Moreover, we consider the reasonable case where the amplitude for an electron to conserve (flip) its spin is $t_{++} = t_{--} = A$ $(t_{-+} = t_{+-} = B)$, with $A \gtrsim B$ (see sub-Section II A 3). In this case we have $J_{++} = J_{+-} = J_{-+} = J_{--} \equiv J$. Finally, if we use the free electron gas relation $k_F^\sigma = \sqrt[3]{6\pi^2 n_\sigma}$ to express the exchange parameter in terms of the average density of hopping electrons in the $\sigma$ spin state, $n_\sigma$, we obtain the result stated in Eqs. (3)-(8) of the main text.

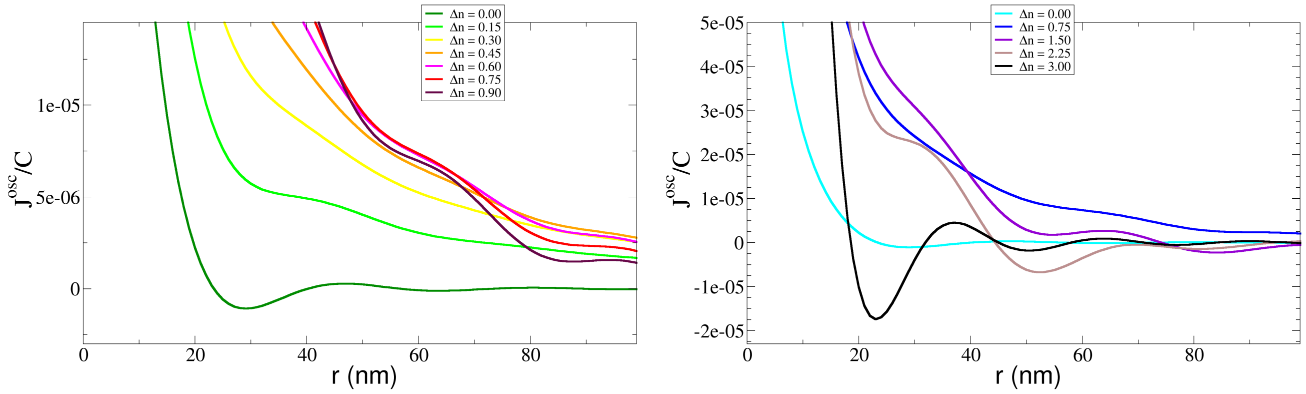


**Supplementary Figure** S3. Plot of the $J(r, n_+, n_-)$ [given by main text's Eq. (3) with $A = 1$ and $B = 0.96$] in terms of the distance between nanoparticles $r$. We plot the $J(r, n_+, n_-)$ for several spin-imbalances $\Delta n = n_+ - n_-$ (expressed in units of $10^{22}$ $\mathrm{m}^{-3}$). In the left panel we plot curves in the range $\Delta n \in [0.0, 0.9] \times 10^{22}$ $\mathrm{m}^{-3}$, while in the right panel we plot curves in the range $\Delta n \in [0.0, 3.0] \times 10^{22}$ $\mathrm{m}^{-3}$. Note that the periodicity of the oscillation of $J$ depends on the spin-imbalance. For example, if the spin-imbalance has a value such that $\Delta n \in [0.15, 0.9] \times 10^{22}$ $\mathrm{m}^{-3}$, then the indirect exchange remains ferromagnetic for at least $r \gtrsim 100$ nm. The constant $C$ dividing $J^{\mathrm{osc}}$ reads $C = J_0\mu_0^2\mu_B^2 m^*/(4(2\pi)^3\hbar^2)$.

In Supplementary Fig. S3 we can see the dependence of the exchange parameter [given by main text's Eqs. (3)-(8) with $A = 1.$ and $B = 0.96$] in the distance $r$, for several spin-imbalances. From it we conclude that the exchange parameter period of oscillation is also strongly dependent on the spin-imbalance. Moreover, for some values of the spin imbalance (namely, $\Delta n \in [0.15, 0.9] \times 10^{22}$ $\mathrm{m}^{-3}$) the exchange coupling remains ferromagnetic for distances greater than 100 nm.

In order to qualitatively account for the average disorder effects (see sub-Section II A 3), we exponentially damp the indirect exchange parameter computed above into $J(r, n_+, n_-) \longrightarrow J(r, n_+, n_-)e^{-r/\xi}$, where in the metallic case, $\xi$ is the electron's mean free path. The stronger the disorder, the stronger the damping of $J(r)$. The strong disorder in our system will render $\xi$ to be small and thus the exponential suppression essentially kills all longer ranged interactions. Only those with distances comparable with the first-neighbor separation will be relevant. In the comparison with the experiment we take $\xi$ to be a fitting parameter comparable to the spacing between the nanoparticles.

### *3. Discussion of the approximations employed in the above calculations*

Several approximations were done to arrive at the result for the electron mediated exchange coupling term written in main text's Eq. (3): we have assumed the interaction between the hopping electrons and the nanoparticles to be local; the variable range hopping character of the hopping electrons was neglected; the effects arising from the system's strong disorder were initially ignored and then qualitatively reintroduced later leading to the exponential damping of the exchange coupling between nanoparticles; in computing the $\mathbf{k}, \mathbf{q}$-sums in Eq. (S6) we have both considered the hopping electrons spectrum to be that of a free electron gas, and the system to be at zero temperature; we have made a particular choice of the electronic spin-flip amplitudes $t_{\sigma\eta}^{(\alpha)}$; and we have considered the nanoparticles' magnetic moments to be parallel oriented Ising moments. Several of these approximations were already discussed above, and thus in the following paragraphs we are going to comment on those not yet discussed.

As referred above, to ignore the strong (position and energy) disorder of the sites at which the electrons can sit, amounts to change the hopping electrons' nature from strongly localized to strongly delocalized. This simplification is equivalent to consider an average over disorder and to assume that the electrons move on a perfect (cubic) lattice. The natural way of describing such a system is in terms of well defined wave-vectors. However, these are only meaningful in the scope of a disorder free problem. Since the system we study is strongly disordered, we opt by expressing the effective

Hamiltonian (computed after the disorder average approximation) in terms of the average density of hopping electrons with spin $\sigma = \pm 1$, $n_\sigma$, than in terms of Fermi wave-vectors, $k_F^\sigma$.

In addition, the exponential damping of the exchange interaction rests upon the works of De Gennes[5], Lerner[6] and Sobota et al.[7]. De Gennes[5] has shown that, in a weakly disordered metal (by a random scalar potential) the average indirect RKKY exchange interaction is exponentially damped at distances greater than the electron mean free path. Using field theoretical techniques Lerner[6] showed that in the strong disorder limit, despite the fact that the average RKKY interaction is exponentially damped at distances greater than the electron mean free path, the magnitude of the actual interaction is strongly dependent on the disorder configuration. In fact it is better characterized by a broad log-normal distribution that indicates that fluctuations that are considerably larger than the typical value of the interaction can indeed occur. In a recent numerical study Sobota et al.[7] found out that strong disorder and localization give rise to a RKKY interaction with a distribution function that develops a strongly non-Gaussian form with long tails. They concluded that the typical value of the interaction is better characterized by the geometric average of this distribution and that this average is exponentially suppressed in the presence of strong localization.

When computing the sums over momentum giving rise to Eq. (S4) and Eq. (S5) we have assumed for simplicity that the energy dispersion of the hopping electrons moving on the perfect cubic lattice (under a first-neighbor tight-binding model) was that of a free electron gas (with spin $\sigma$) with an effective mass $m^*$, $E_\sigma(\mathbf{k}) = \hbar^2 \left(\mathbf{k}^\sigma\right)^2/(2m^*)$. It is acceptable to do such an approximation for electrons on a 3D lattice if the system's Fermi level is at the *bottom* of the co-sinusoidal band, i.e. if $k_F^\sigma a \ll 1$, where $a$ is the cubic lattice spacing. Despite the difficulty in estimating such a lattice spacing, we consider the reasonable value of 1 nm. Then, the estimate of the density of hopping electrons with spin $\sigma$ made in sub-Section I A, gives $k_F^\sigma \lesssim 0.12$ nm$^{-1}$, which indicates that the free electron gas approximation is reasonable.

The zero-temperature approximation employed in the computation of the sums over momentum giving rise to Eq. (S4) and Eq. (S5), greatly simplifies the sum argument by eliminating the Fermi-Dirac factor while imposing a cut-off on the sum. However, it is only reasonable when $k_B T \ll E_F = \hbar^2 k_F^2/(2m^*)$. In our case, the condition $k_B T \ll E_F$ holds if the free electron gas effective mass, $m^*$, is at least two orders of magnitude smaller than the bare electron mass (since $T = 300$ K and $k_F \approx 0.12$ nm$^{-1}$). Kim et al.[12,13] have computed the RKKY interaction (without spin-flips) for finite temperature and found out that it decreases both the magnitude of the interaction and its period of oscillation between positive and negative values. To extend this computation to the case where electron spin-flips are present would deserve a publication of its own. However, we do not think that temperature is going to make the $J(r)$ not clearly ferromagnetic for first-neighbor distances, since in Supplementary Fig. S3 we can see that there are big ranges of spin-imbalances (for example, $0.15 \times 10^{22} \lesssim n_+ - n_- \lesssim 1.50 \times 10^{22}$ electrons/m$^3$) for which the exchange coupling is ferromagnetic (i. e. $J(r) > 0$) up to distances between nanoparticles of $\gtrsim 70$ nm.

Remember that in order to write main text's Eq. (3), we have both considered that the hopping electrons' spin-flip amplitudes at different nanoparticles are all equal, and that the amplitude for an electron to conserve its spin, $t_{++} = t_{--} = A$, is slightly bigger than the amplitude for it to have its spin flipped, $t_{-+} = t_{+-} = B$. This simplification is equivalent to consider that the spin-flip amplitudes are decoupled from the nanoparticles at which they occur, namely, from their magnetic moment's magnitude and orientation. This can be regarded as a first order approximation to the problem that should give the global tendency of the system's magnetic behavior.

Finally, let us briefly comment on the reasonability of considering a model where the nanoparticles' magnetic moments are parallel oriented Ising moments. Recall that the nanoparticles' magnetic moments can be viewed as Heisenberg moments that are constrained to point around their randomly oriented easy axis. We can ignore fluctuations around their ground states and thus assume them to be Ising moments aligned (parallel or anti-parallel) with their randomly oriented easy axis. In such a case, a generalization of the previous calculation implies that we start by modifying the model's microscopic Hamiltonian in Eq. (S2) so that the Ising moments can have their easy axis arbitrarily aligned. This amounts to consider all the terms in the dot product $\hat{\mathbf{S}}_i \cdot \mathbf{M}_\alpha = m_\alpha \left(\hat{S}_i^x \sin(\theta_\alpha)\cos(\gamma_\alpha) + \hat{S}_i^z \sin(\theta_\alpha)\sin(\gamma_\alpha) + \hat{S}_i^z \cos(\theta_\alpha)\right)$ so that the microscopic Hamiltonian in Eq. (S2) is modified into

$$\hat{H} = -\sum_{ij}\sum_{\sigma_i,\sigma_j}\left(\gamma_{ij}\delta_{\sigma_i\sigma_j}\hat{c}^\dagger_{i\sigma_i}\hat{c}_{j\sigma_j} + \text{h.c.}\right) - J_0\mu_0\mu_B^2\sum_{i\alpha}\sum_{\sigma_i} m_\alpha \delta(\mathbf{r_i}-\mathbf{r}_\alpha)\Bigg[\Big(\sigma_i\cos(\theta_\alpha)\,t^{(\alpha)}_{\sigma_i\sigma_i} + \sin(\theta_\alpha)\,e^{-i\sigma_i\gamma_\alpha}\,t^{(\alpha)}_{-\sigma_i\sigma_i}\Big)\hat{c}^\dagger_{i\sigma_i}\hat{c}_{i\sigma_i} + \Big(\sin(\theta_\alpha)\,e^{i\sigma_i\gamma_\alpha}\,t^{(\alpha)}_{\sigma_i\sigma_i} - \sigma_i\cos(\theta_\alpha)\,t^{(\alpha)}_{-\sigma_i\sigma_i}\Big)\hat{c}^\dagger_{i,-\sigma_i}\hat{c}_{i\sigma_i}\Bigg], \qquad \text{(S8)}$$

where the angles $\theta_\alpha \in [0,\pi]$ and $\gamma_\alpha \in [0, 2\pi]$ define the orientation of its Ising moment. The spin operator of a hopping electron sitting at $\mathbf{r}_i$, was written as $\hat{\mathbf{S}}_i = (\hat{S}_i^x, \hat{S}_i^y, \hat{S}_i^z) = \mu_B \sum_{\sigma'\sigma} \hat{c}^\dagger_{i\sigma_i}\left([\sigma^x]_{\sigma_i\sigma_i'}, [\sigma^y]_{\sigma_i\sigma_i'}, [\sigma^z]_{\sigma_i\sigma_i'}\right)\hat{c}_{i\sigma_i'}$, where $\sigma^x$, $\sigma^y$ and $\sigma^z$ stand for the Pauli matrices, while $\sigma_i = \pm 1$ indicates the electronic spin orientation ($\sigma^z$ eigenvalue) of the electron sitting at $\mathbf{r}_i$.

Note that now, due to the presence of $\hat{\sigma}_i^x$ and $\hat{\sigma}_i^y$, the effective terms conserving and flipping the hopping electron's spin (when these interact with the nanoparticles) are different from those obtained in the case where all the nanoparticles' easy axis are parallel – see Eq. (S2). Naturally, the interaction between the hopping electrons and the nanoparticles is now going to also depend on the orientation of the nanoparticle's magnetic moment, which is going to affect the polarization of the electron sea surrounding the nanoparticle. Therefore, the indirect exchange coupling between two nanoparticles is necessarily going to be a function of their orientation.

By integrating the hopping electrons degrees of freedom we end up with a slightly different indirect exchange parameter between two nanoparticles with magnetic moments given by $\mathbf{M}_\alpha = m_\alpha\left(\sin\theta_\alpha\cos\gamma_\alpha, \sin\theta_\alpha\sin\gamma_\alpha, \cos\theta_\alpha\right)$ and $\mathbf{M}_\beta = m_\beta\left(\sin\theta_\beta\cos\gamma_\beta, \sin\theta_\beta\sin\gamma_\beta, \cos\theta_\beta\right)$. Such an indirect exchange will then read $J(r, n_+, n_-; \gamma_\alpha, \theta_\alpha, \gamma_\beta, \theta_\alpha) =$

$C\sum_{\sigma=\pm}\left(\mathbb{X}_{\sigma}^{(\alpha\beta)}\,\mathbb{J}^{(1)}(r,n_{\sigma})+\mathbb{W}_{\sigma}^{(\alpha\beta)}\,\mathbb{G}(r,n_{+},n_{-})\right)$, where $C\equiv J_0^2\mu_0^2\mu_B^2 m^*/(32\pi^3\hbar^2)$, $\mathbb{G}(r,n_{+},n_{-})$ is again given by the expression written in main text's Eqs. (4)-(8), while the functions $\mathbb{X}_{\sigma}^{(\alpha\beta)}$ and $\mathbb{W}_{\sigma}^{(\alpha\beta)}$ read

$$\mathbb{X}_{\sigma}^{(\alpha\beta)}\equiv\mathbb{X}_{\sigma}(\gamma_\alpha,\theta_\alpha,\gamma_\beta,\theta_\alpha)=\left[\sigma\cos\theta_\alpha t_{\sigma\sigma}^{(\alpha)}+e^{-i\sigma\gamma_\alpha}\sin\theta_\alpha t_{-\sigma,\sigma}^{(\alpha)}\right]\left[\sigma\cos\theta_\beta t_{\sigma\sigma}^{(\beta)}+e^{-i\sigma\gamma_\beta}\sin\theta_\beta t_{-\sigma,\sigma}^{(\beta)}\right], \tag{S9a}$$

$$\mathbb{W}_{\sigma}^{(\alpha\beta)}\equiv\mathbb{W}_{\sigma}(\gamma_\alpha,\theta_\alpha,\gamma_\beta,\theta_\alpha)=\left[e^{i\sigma\gamma_\alpha}\sin\theta_\alpha t_{\sigma\sigma}^{(\alpha)}-\sigma\cos\theta_\alpha t_{-\sigma,\sigma}^{(\alpha)}\right]\left[e^{-i\sigma\gamma_\beta}\sin\theta_\beta t_{-\sigma,-\sigma}^{(\beta)}+\sigma\cos\theta_\beta t_{\sigma,-\sigma}^{(\beta)}\right]. \tag{S9b}$$

In order to gain some insight on this expression we sampled it for two nanoparticles at a distance of $r=12$ nm, arbitrary orientations and several spin-imbalances. The value of $J(r,n_+,n_-;\gamma_\alpha,\theta_\alpha,\gamma_\beta,\theta_\alpha)$ is naturally dependent on the angle between the two magnetic moments, $\varphi_{\alpha\beta}$, taking values in a given interval for a particular $\varphi_{\alpha\beta}$ depending on the orientation of the two Ising moments relatively to the hopping electrons spin polarization direction. Averaging the sampled values of $J(r,n_+,n_-;\gamma_\alpha,\theta_\alpha,\gamma_\beta,\theta_\alpha)$ and plotting them in terms of the angle $\varphi_{\alpha\beta}$ (see Supplementary Fig. S4) we conclude that the average value of $J(r,n_+,n_-,\varphi_{\alpha\beta})$ is going to be ferromagnetic at typical inter-nanoparticle distances (i. e., $r\approx 12$ nm), with its magnitude increasing with increasing spin-imbalance – see panel (a) of Supplementary Fig. S4. Moreover, it will decay and oscillate with the inter-nanoparticle distance – see panel (b) of Supplementary Fig. S4. This is a strong indication that such a system will qualitatively behave in a very similar manner to that of the model

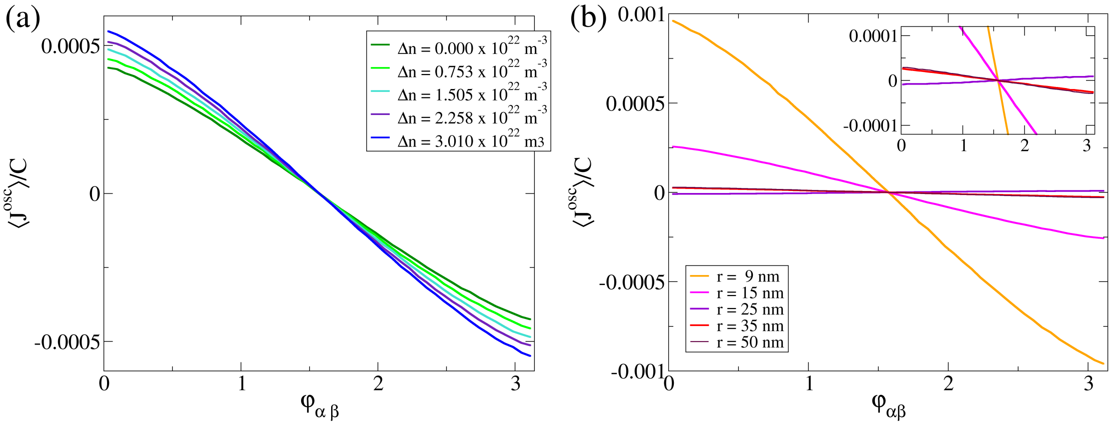


**Supplementary Figure** S4. **Average indirect exchange between non-parallel Ising moments.** Plot of the oscillating part of the average indirect exchange in terms of the angle between the two nanoparticles. **(a)** Comparison between the average of the oscillating part of $J$ between two nanoparticles at $r=12$ nm for different spin-imbalances. **(b)** Comparison between the average of the oscillating part of $J$ between two nanoparticles (under a spin-imbalance of $\Delta n=2.258\times10^{22}$ m$^{-3}$) at different distances. The inset is a zoom in around $\langle J^{osc}\rangle/C\approx 0$.

considered in the main text (parallel Ising moments), thus justifying that simpler approach over this one.

### B. Monte Carlo simulations

Let us start by pointing out that the experimental results strongly suggest that the first order term of main text's Eq. (2) plays a secondary role on the genesis of the magnetic state of the nanocomposite. The fact that the left panel of Supplementary Fig. S2 shows that the nanocomposite's hysteretic response to an external magnetic field depends on the initialization field (i. e., on the spin-imbalance), clearly demonstrates that the second order term dominates over the first order one. Note that the first order term can be seen as a local effective magnetic field proportional to the spin-imbalance of hopping electrons surrounding a nanoparticle. If, fixing the temperature, the first order term was the dominant one, then the two hysteretic curves (obtained from systems initialized with different magnetic fields, i. e., different spin-imbalances) would only differ on their initial part, collapsing into each other thereafter. In a system of independent nanoparticles' magnetic moments, the hopping electrons' spin-imbalance should be tightly linked to the system's magnetization (see Section III). Therefore, whenever the nanocomposite's magnetization is saturated by a sufficiently strong magnetic field, the spin-imbalance should also increase to a maximum (saturated) value. Accordingly, if two such systems (each one of them sustaining distinct spin-imbalances at an initial time) were subjected to a strong magnetic field saturating their magnetization, then their spin-imbalances would be brought to similar values and from then onward they would present a similar hysteresis. This does not happen if the system is controlled by the second order term, since its local spin-imbalance will be preserved by the intrinsic magnetization (originating from the coupling between nanoparticles) of the nanocomposite's magnetic domains.

To have a first order term that is negligible when compared with the second order one [multiplied by the exponential damping factor – see main text's Eq. (2)] is perfectly compatible with the theoretical model. Despite the fact that these two terms are obtained after expanding on the coupling constant [see Eq. (S2)] and integrating the electronic degrees of freedom, their relative magnitude is determined by the external parameters: spin-imbalance, $n_+-n_-$; distance between the interacting nanoparticles, $r$; magnitude of the nanoparticles' magnetic moments; strength of the exponential suppression of the RKKY interaction due to disorder, $\xi$; scale of the hopping electron-nanoparticle interaction, $J_0$; effective mass parameter of the model, $m^*$; electron spin-flip amplitudes $A$ and $B$. Interestingly, whenever the chosen parameters are compatible with the logarithm series expansion [see Eq. (S3)], i. e., when they fulfill the condition

$\gamma_{ij} \gg J_0\mu_0\mu_B^2 m_\alpha t^{(\alpha)}_{\eta_i\sigma_i}$, for all $i, j, \alpha, \eta_i, \sigma_i$, there is always a region of the external parameter space for which the first order term is irrelevant when compared with the second order one.

Therefore, from here onward we will consider that the indirect exchange term dominates the system's physics. With the intent to check if such model holds as a suitable explanation for the tunability of the nanocomposite's ordering temperature upon manipulation of its spin-imbalance (see main text's Fig. 2), we have performed Monte Carlo simulations of the following model: randomly positioned Ising moments in 3 dimensions with random magnitudes uniformly distributed around 4 $\mu_B$; these moments interact via an exchange coupling, given by main text's Eqs. (3)-(8) with $A = 1.00$ and $B = 0.96$. The free parameters of the system are: the electron gas effective mass, $m^*$; the characteristic length controlling the exponential damping of the exchange coupling, $\xi$; and the constant $J_0$ controlling the scale of the electron-nanoparticle interaction. We keep $m^*$ at all times equal to $100\, m_e$, where $m_e$ stands for the bare electron mass, using $\xi$ and $J_0$ as fit parameters that will be fixed by the requirement that the dependence of the ordering temperature with spin-imbalance obtained from the Monte Carlo simulations reproduces the experimental one – see main text's Fig. 4(c).

Qualitatively, we expect that for values of $\xi$ smaller than the inter-nanoparticle distance ($r \approx 12$ nm) the exponentially damped coupling should give rise to the ordering of the system in magnetic clusters that interact weakly between themselves. Upon decreasing temperature, the magnetic moments inside each cluster align, with different clusters doing so at slightly distinct temperatures. As clusters interact weakly, individual clusters will generally have different magnetization directions. As a consequence, the system should not in general present long-range order when temperature is decreased below the *blocking* temperature $T_b$. We have confirmed this by Monte Carlo simulations using the Cluster algorithm[14].

However, if we start from an ordered state generated, for example, by applying an external magnetic field when the spin-imbalance is being generated (as is done in the experiment), then long-range order should be observed since the nearly independent clusters were from the beginning aligned by the external magnetic field. We have used Metropolis Monte Carlo algorithm[15] to investigate this.

The use of Metropolis Monte Carlo at very low temperatures is highly inefficient in exploring the complete phase space of the system. At low temperatures the single-flip Monte Carlo dynamics cannot escape from the region of the phase space around a local energy minimum. A random initial configuration will very fast relax to its nearest local energy minimum and get stuck there *ad eternum*. This is an extreme form of critical slowing down that is characteristic of single spin-flip Metropolis dynamics (but that is avoided by the Cluster algorithm dynamics).

To use Metropolis single-flip dynamics starting from an initial configuration very close to the global energy minimum will thus imply that at low temperatures the Monte Carlo sweep will only explore the phase space region around the global minimum. Increasing the temperature will allow the Monte Carlo sweep to explore increasingly larger regions of the phase space. Therefore, Monte Carlo averages (using this dynamics) at low temperatures will not result in correct statistical averages (indicative of the existence of spontaneous phase transitions) due to the partial exploration of the phase space. However, such averages will nevertheless give a good indication of the phase space accessible to the system (or else, of the low-temperature system's magnetic state) when a magnetic field is initially applied to the sample (as is done in the experiment) ordering most of its independent clusters in the same direction.

In main text's Fig. 4(b) we demonstrate exactly this: when starting from an ordered state, single-flip Metropolis Monte Carlo simulations suggests that a long range ordered state is possible below a given blocking temperature, $T_b$. Moreover, this blocking temperature is shown to depend on the magnitude of the indirect exchange, that we know is dependent on the spin-imbalance of the hopping electrons. In main text's Fig. 4(c) we compare the dependence of $T_b$ with the spin-imbalance measured in the experiment and the dependence arising from the numerical simulations (with $\xi = 5$ nm and $J_0 = 2.2 \times 10^6$).

Finally, note that in these Monte Carlo studies we have investigated the temperature dependence of the sample magnetization (after an initial magnetic field was applied to it). We are at all temperatures using the same temperature-independent ($T = 0$) expression for the indirect exchange coupling. However, as we have previously referred, the exchange coupling should depend on temperature – see discussion of sub-Section II A 3. In particular, as we know that increasing temperature decreases the magnitude of the indirect coupling computed at $T = 0$,[12,13] then, if temperature would be taken into account in computing the indirect exchange of main text's Eqs. (3)-(8), we expect that the numerical results would show a less pronounced slope in main text's Fig. 4(c).

## III. AVENUES FOR FUTURE INVESTIGATIONS

Although beyond the scope of the current work, here we discuss some aspects of this novel system that deserve future investigation, namely related to the spin-imbalance generation and the magnetization measurements.

### The spin-imbalance generation

The hopping electrons' spin-imbalance is generated by the *initialization* process where we use an external magnetic field to drive the magnetic orientation of the two cobalt electrodes that control the flow of the electric current across the device (at room temperature). We expect the biggest spin-imbalance to occur for the case where the electrodes are perfectly anti-parallel aligned, i. e. at $B_{\text{ext}} \approx 0$. However, as seen in main text's Fig. 2(b), the highest spin-imbalance occurs for an *initialization* process done with $B_{\text{ext}} \gtrsim 0.04$ T, when the second electrode is already partially reversed [see main text's Fig. 3(b)].

An explanation for this observation invokes the combined action of the magnetic field applied during the *initialization* process, $B_{\text{ext}}$, and the (spin-imbalance dependent) indirect exchange interaction between the nanoparticles. When $B_{\text{ext}} = 0$ T, the electrodes are anti-parallel aligned and, if no other factor would be playing a role, we would expect that the spin-imbalance generated would be maximal. However, recall that the hopping electrons' are prone to have their spin flipped when interacting with the system's nanoparticles, especially at room-temperature. When no magnetic

field is being applied, the nanoparticles' clusters are oriented in random directions, no global magnetization exists in the system, and then nothing counteracts the thermal activated electron spin-flips. As a consequence, the hopping electron's spin-imbalance will progressively vanish in the electrons' path between the source and the drain electrode. This would explain the observation of a low capacitance for anti-parallel electrodes and $B_{\rm ext} \approx 0$ T.

When we *initialize* the system with a non-zero $B_{\rm ext}$, the thermal spin-flips of the hopping electrons will be progressively counterbalanced by the action of the nanocomposite's magnetization (arising from the combined action of the indirect exchange coupling, magnetizing each cluster of nanoparticles, and the external magnetic field, orienting the different clusters in a given direction), since the hopping electrons will rather align along the direction of magnetization of the nanocomposite. The competition between the spin-flips and the effect of the nanocomposite's magnetization will thus determine the measured spin-imbalance.

It is thus reasonable to expect that a stronger magnetic field generates a greater spin-imbalance. Note however that stronger magnetic fields also lead to electrodes that are not completely anti-parallel, and thus to a smaller *potential* spin-imbalance. This explains why the maximal spin-imbalance is not at $B_{\rm ext} \lesssim 0.05$ T (immediately before the electrodes become parallel), but instead somewhere in between this value and $B_{\rm ext} = 0$ T.

## The spin-imbalance after initialization

It is likely that the capacitance (measured during the *initialization* process) is not going to exactly correspond to the actual capacitance of the nanocomposite after the *initialization* process is terminated and the system relaxes to equilibrium (through thermal activated spin-flips of the hopping electrons). In fact, the measured capacitance is going to fix an upper bound on the spin-imbalance of the nanocomposite. Therefore, one other relevant question to ask is how the spin-imbalance relaxes to its equilibrium value after the *initialization* process is finished, i. e. after the *initialization* current and magnetic field are turned off.

We expect that the spin-imbalance equilibrium value originates from a competition between the thermal activated electron spin-flips and the nanocomposite's magnetization after the magnetic field is turned off. If the magnetization is zero, the spin-imbalance should completely vanish since nothing counterbalances the action of the thermal activated spin-flips of the hopping electrons. However, if the nanocomposite is magnetized, an equilibrium situation should be achieved where the action of the thermal activated spin-flips and the counteraction of the nanocomposite's magnetization cause the electron's spin-imbalance to relax to a non-zero value. This is one of the reasons for the big horizontal error bars in the experimental points of main text's Fig. 4(c).

## The role of the initialization magnetic field

As referred in sub-Section II B, the system's strong disorder gives rise to a short-range exponential damping of the electron mediated indirect exchange between nanoparticles. This produces an ordering of the nanoparticles in nearly independent magnetic clusters with distinct ordering temperatures (that depend on the distances between them and the strength of the exchange coupling), and thus no spontaneous long-range magnetic order is expected upon decreasing temperature. However, when an external magnetic field is applied to the system during the *initialization* process (as done in the experiment), we expect that in average the nearly independent clusters will end up oriented along the same direction. Thus, after the *initialization* magnetic field is turned off, the magnetic ordering should be preserved as long as the coupling between nanoparticles does not vanish, i. e. as long as the spin-imbalance survives.

In trying to test this hypothesis we have found that no ferromagnetism is observed in the nanocomposite if no magnetic field is applied to the system when the spin-polarized current is flowing across the device. However, we must stress that this observation is not a definitive proof that the system is arranged in magnetic clusters. It can also be due to the two issues discussed just above: either to the fact that in the absence of a magnetic field the electrodes' configuration may not be able to generate a sufficiently big spin-imbalance so that the nanoparticles become ferromagnetically coupled; or to the fact that no spin-imbalance can be sustained if the nanocomposite is in a zero magnetization state (see above), and thus the indirect coupling between nanoparticles should rapidly vanish after the *initialization*.

## The magnetization vs. temperature curves

We are now going to further comment on the special case of the maroon magnetization curve in main text's Fig. 2(a) of the main text. After what we have just said about the conditions that can generate and preserve a non-zero spin-imbalance in the nanocomposite, the maroon curve in main text's Fig. 2(a) is puzzling: no magnetization is seen at room temperature, but only for $T < 280$ K.

If immediately after the *initialization* process is terminated, the generated spin-imbalance is not sufficiently strong to sustain a macroscopic magnetization (as experimentally observed), then we expect the spin-imbalance to rapidly and completely vanish. If the spin-imbalance is nearly zero, then we expect that no macroscopic magnetization would ever be observed upon decreasing the temperature (provided we do not go to sufficiently low temperatures as to make the magnetostatic interaction between nanoparticles relevant).

A possible explanation for such an observation ascribes to the electrodes the responsibility for this phenomenon. Upon the application of a $B_{\rm ext} = 0.02$ T during the *initialization*, the electrodes' configuration does not give rise to a sufficiently big spin-imbalance so as to generate long range order. Therefore, immediately after the end of the *initialization* process, the spin-imbalance vanishes everywhere except around the electrodes. The local magnetic field produced by the anti-parallel ferromagnetic electrodes generates a spin-imbalance in their close vicinity. If the electrodes are not perfectly anti-parallel (since the $B_{\rm ext} = 0.02$ T applied during the *initialization* partially reversed one of the electrodes), then it is possible that upon decreasing temperature this residual spin-imbalance gives rise to a sufficiently strong coupling between the nanoparticles so that a magnetization is observed.

## High temperatures and the magnetization vs. temperature curves

Frequently, after recording the system's magnetization from $T = 230$ K up to $T = 330$ K (where the nanocomposite's magnetization vanishes), if then the temperature is decreased while the magnetization is being recorded, we see that the magnetization curve is either shifted to lower temperatures or it is completely eliminated. Two phenomena may be contributing to this: the enhanced graphene oxide reduction at higher temperatures; and the vanishing of any spin-imbalance as the magnetization becomes zero at $T > T_b$.

The temperature enhanced graphene oxide reduction will have as a consequence that the hopping electrons will visit the nanoparticles less often since they have a lot more sites available at the graphene oxide. This is going to make the indirect exchange between nanoparticles weaker, which will then lower the ordering temperature. An indication that this phenomena is occurring is the fact that after the increase in temperature the system's conductivity often increases several fold, indicating that the graphene oxide reduction is relevant.

Also, and likely more important, when temperature is increased above the blocking temperature so that the nanocomposite's demagnetizes, then the spin-imbalance should rapidly vanish since no magnetization exists to counterbalance the thermal activated flipping of the hopping electrons'. As a consequence, since there is no spin-imbalance and thus the nanoparticles are independent, then no magnetization should be observed upon temperature decrease.

## Role of graphene oxide and its degree of oxidation

As mentioned in the main text, the graphene oxide used in the nanocomposite is highly defective and partially reduced (between 18% and 20%). Studies have been done where the nanocomposite's graphene oxide was substituted by other poorly conducting media such as the non-conducting form of Polyaniline[3], and no ferromagnetism was observed. Nanocomposites with completely oxidized (and highly defective) graphene oxide were also studied[16] and again no ferromagnetism was observed. We thus may speculate that the difference between these results and the ones presented here is linked to the additional paramagnetic centers present on the highly defective and partially reduced graphene oxide flakes (which are absent or less frequent on the other poorly conducting materials tested so far). Likely the hopping electrons will also interact with these additional paramagnetic centers and therefore will effectively couple them to the iron oxide nanoparticles' magnetic moments. This may favour the percolation of magnetic ordering in between magnetic clusters facilitating long-range magnetic order on the nanocomposite.

Note in addition that the experimental observations are rather sensitive to the degree of reduction of the graphene oxide. As mentioned above, when the device is subjected to moderately high temperatures the graphene oxide flakes are usually reduced to such an extent that the ferromagnetic state is irreversibly lost. Despite the obvious complications that such phenomenon poses, it can also be regarded as potentially interesting: the easy tunability of graphene oxide's degree of reduction (though unidirectional/irreversible) can be used to manipulate the magnitude and nature of the coupling between magnetic moments of the nanocomposite.

# REFERENCES


1. Fleetwood, D. M. *et al.* "Effects of oxide traps, interface traps, and 'border traps' on metal-oxide-semiconductor devices", *J. Appl. Phys.* **73**, 5058–5074 (1993).
2. McCarthy, K. T., Hebard, A. F. & Arnason, S. B. "Magnetocapacitance: Probe of spin-dependent potentials", *Phys. Rev. Lett.* **90**, 117201 (2003).
3. Lin, A. L., Wu, T., Chen, W. & Wee, A. T. S. "Room temperature positive magnetoresistance via charge trapping in polyaniline-iron oxide nanoparticle composites", *Appl. Phys. Lett.* **103** (2013).
4. Zhu, J. *et al.* "Magnetic field induced capacitance enhancement in graphene and magnetic graphene nanocomposites", *Energy Environ. Sci.* **6**, 194–204 (2013).
5. De Gennes, P.G. "Polarisation de charge (ou de spin) au voisinage d'une impuret dans un alliage", *J. Phys. Radium* **23**, 630–636 (1962).
6. Lerner, I. V. "Dependence of the ruderman-kittel-kasuya-yosida interaction on nonmagnetic disorder", *Phys. Rev. B* **48**, 9462–9477 (1993).
7. Sobota, J. A., Tanasković, D. & Dobrosavljević, V. "Rkky interactions in the regime of strong localization", *Phys. Rev. B* **76**, 245106 (2007).
8. Ruderman, M. A. & Kittel, C. "Indirect exchange coupling of nuclear magnetic moments by conduction electrons", *Phys. Rev.* **96**, 99–102 (1954).
9. Kasuya, T. "A theory of metallic ferro- and antiferromagnetism on zener's model", *Prog. Theo. Phys.* **16**, 45–57 (1956).
10. Yosida, K. "Magnetic properties of cu-mn alloys", *Phys. Rev.* **106**, 893–898 (1957).
11. Fullenbaum, M. S. & Falk, D. S. "Electron spin polarization due to a magnetic impurity", *Phys. Rev.* **157**, 452–456 (1967).
12. Kim, J. G., Lee, E. K. & Lee, S. "Temperature-dependent ruderman-kittel-kasuya-yosida interaction", *Phys. Rev. B* **51**, 670–673 (1995).
13. Kim, J. G., Choi, Y.-H. S., Lee, E. K. & Lee, S. "Temperature-dependent free-electron susceptibility for one, two, and three dimensions", *Phys. Rev. B* **59**, 3661–3670 (1999).
14. Wolff, U. "Collective monte carlo updating for spin systems", *Phys. Rev. Lett.* **62**, 361–364 (1989).
15. Metropolis, N., Rosenbluth, A. W., Rosenbluth, M. N., Teller, A. H. & Teller, E. "Equation of state calculations by fast computing machines", *J. Chem. Phys.* **21**, 1087–1092 (1953).
16. Lin, A. L. *et al.* "Room temperature magnetic graphene oxide- iron oxide nanocomposite based magnetoresistive random access memory devices via spin-dependent trapping of electrons", *Small* **10**, 1945–1952 (2014).
